# Genetic Pathways of Aging and Their Relevance in the Dog as a Natural Model of Human Aging

**DOI:** 10.3389/fgene.2019.00948

**Published:** 2019-10-18

**Authors:** Sára Sándor, Enikő Kubinyi

**Affiliations:** Department of Ethology, Eötvös Loránd University, Budapest, Hungary

**Keywords:** hallmarks of aging, animal aging models, family dogs, aging genetics, dementia research

## Abstract

Aging research has experienced a burst of scientific efforts in the last decades as the growing ratio of elderly people has begun to pose an increased burden on the healthcare and pension systems of developed countries. Although many breakthroughs have been reported in understanding the cellular mechanisms of aging, the intrinsic and extrinsic factors that contribute to senescence on higher biological levels are still barely understood. The dog, *Canis familiaris*, has already served as a valuable model of human physiology and disease. The possible role the dog could play in aging research is still an open question, although utilization of dogs may hold great promises as they naturally develop age-related cognitive decline, with behavioral and histological characteristics very similar to those of humans. In this regard, family dogs may possess unmatched potentials as models for investigations on the complex interactions between environmental, behavioral, and genetic factors that determine the course of aging. In this review, we summarize the known genetic pathways in aging and their relevance in dogs, putting emphasis on the yet barely described nature of certain aging pathways in canines. Reasons for highlighting the dog as a future aging and gerontology model are also discussed, ranging from its unique evolutionary path shared with humans, its social skills, and the fact that family dogs live together with their owners, and are being exposed to the same environmental effects.

## Introduction

Dogs (*Canis familiaris*) are special in the animal kingdom in many aspects. Being the oldest domesticated species, they accompany humans for approximately 15,000–100,000 years (estimates depend on the different approaches used to study their origin; see Vila, 1997; Larson et al., 2012; Thalmann et al., 2013; Frantz et al., 2016; Botigué et al., 2017). Consequently, they have adapted to the special social environment of human communities in a way unmatched by any other species and thus earned their rightful title as man’s best friend. They have also gained diverse functionality throughout the millennia, which resulted in a phenotypic variability unrivalled by any other species. Recently, the species has also been promoted to be a model of human physiology and disease. The sequencing of the dog genome (Lindblad-Toh et al., 2005) and the development of high-resolution genotyping arrays to support genome wide association studies (GWAS) (Hayward et al., 2016) were important steps to open up new perspectives of genetic investigations in dogs. Recently, a growing number of full genome sequences from various dog breeds have also been added to the genetic toolkit of canine researchers (Dreger et al., 2016a; Dreger et al., 2016b; Kim et al., 2018). The importance of these improvements is clearly visible through the expanding list of reported disease associated polymorphisms in dogs. Examples like the famous case of the narcolepsy causing mutation in the canine *Hypocretin Receptor 2* gene (Lin et al., 1999), which turned the focus of researchers to its human homolog’s role in narcolepsy, have demonstrated how canine genetics can benefit humans.

Genetic analyses have also helped to shed light on the origin and evolution of dogs and on the divergence of breeds (vonHoldt et al., 2010; Axelsson et al., 2013; Skoglund et al., 2015; Frantz et al., 2016; Freedman et al., 2016; Wang et al., 2016) and have revealed numerous genetic variants responsible for their phenotypic variability. For example, several genes have been shown to affect the body size variability of dogs (Sutter et al., 2007; Hoopes et al., 2012; Rimbault et al., 2013; Plassais et al., 2017), which is unmatched by any other mammalian species. Importantly, dogs also show marked differences in their expected lifespan in connection with body mass. On average, giant sized breeds (above 50 kg) have an expected lifespan of 6–8 years, while small sized breeds (below 10 kg) can live up to 14–16 years (Jimenez, 2016).

This wide range of expected lifespans, together with other aspects, has made dogs promising as model organisms for aging research (Gilmore and Greer, 2015; Creevy et al., 2016; Kaeberlein et al., 2016; Mazzatenta et al., 2017; Hoffman et al., 2018). In this regard, family dogs living as animal companions with their owners could be even more relevant than laboratory dogs (Kaeberlein, 2016). Although laboratory dogs had been traditionally used for a wide range of investigations, including aging research (Cotman and Head, 2008), they have some major limitations. For example, they experience a less complex nutritional and environmental history during their life course than family dogs do, which could lead to major deviances in their base level behavioral parameters. Also, they usually represent only a few breeds. These aspects clearly reduce the power of laboratory dogs to correspond with highly variable natural populations, especially when we consider the range of different aging phenotypes. Actually, describing and characterizing human aging phenotypes is a main goal of researchers, as such variability leads to fundamental differences in individual courses of aging. Living a long life with poor health can negatively affect the welfare of both the elderly and their surroundings. Age-related dementia can especially make a major impact in this regard, rendering patients unable to live an independent life. Therefore, lifespan and healthspan are considered partly independent attributes of human aging, leading to the distinction between healthy aging and pathological aging. Furthermore, in respect to cognitive decline, which can hinder welfare even if no other diseases are present, some authors suggested to discriminate successful aging as a subtype of healthy aging, which is characterized by maintained ability to live an autonomous life until death (Rowe and Kahn, 1987; Rowe and Kahn, 2015). In this regard, family dogs clearly surpass laboratory dogs as models, because they are more abundant, are more variable, and are much more likely to reach an old age and to encounter various aging courses. Although the detailed phenotypic categorization of aging in dogs will require further efforts, definitions for frailty, for example, have already been proposed in the species (Hua et al., 2016).

Nevertheless, it still remains a question how exactly dogs, especially family dogs, can fit in the puzzle of aging genetics among many already well-established experimental models. Despite the huge progress in understanding the genetic basis of morphological variability of dogs, still very little is known about the functional relevance of canine homologs of conserved longevity genes. Currently, this may stand as an obstacle in the way of effectively utilizing dogs as aging models. As family dogs can provide unique insights into many aspects of human aging, the current lack of detailed information about the canine genetic pathways of aging should be overcome by future research approaches. In this review, we provide an overview of the evolutionary conserved biological mechanisms that contribute to aging, following the classification system proposed by López-Otín et al., 2013, and we summarize current knowledge about these pathways in dogs. We also briefly discuss the benefits and limitations of family dogs in aging research and propose possible future directions for canine aging genetic studies.

## Dogs as Model Animals in Aging Research

A plethora of different species has been involved in aging studies to unravel the genetic factors behind this complex biological process. Due to their short lifespan and easy handling under laboratory conditions, the yeast (*Saccharomyces cerevisiae*), the nematode worm *Caenorhabditis elegans*, the fruit fly (*Drosophila melanogaster*), and rodents (mice: *Mus musculus* and rats: *Rattus norvegicus*) all became important contributors to the discovery of longevity affecting genes. Recently, the turquoise killifish (*Nothobranchius furzeri*) has also been added to this palette (Hu and Brunet, 2018). The applicability of various genetic approaches (e.g., induced mutagenesis, RNA interference, gene trapping) in these organisms allowed researchers to specifically target genes for further investigations or to efficiently search for phenotype–genotype associations in mutants.

As most of the revealed pathways turned out to be highly conserved, findings made on model organisms seemed translatable to humans in most cases. However, human aging has characteristics, like the occurrence of age-related dementia, which do not have counterparts in many model organisms. Although this limitation has been overcome by different techniques to induce neurodegenerative processes in the central nervous system (CNS) of model animals, the findings of such studies may not be easily implemented in humans (Jucker, 2010; Puzzo et al., 2015; De Felice and Munoz, 2016). In addition, the interaction between genetic factors and environmental conditions can also vary in humans, meaning that certain variants can have beneficial effects in one context and adverse effects in another (Ukraintseva et al., 2016).

Therefore, studies on human populations are inevitable to understand human aging in its full complexity. Apparently, in this case the genetic toolkit is reduced to associative approaches. With the advent of the genomic era, this has become less of a problem, and several genome wide association studies (GWAS) have actually reported lifespan affecting loci in humans (Deelen et al., 2011; Nebel et al., 2011; Sebastiani et al., 2012; Sebastiani and Perls, 2012; Beekman et al., 2013). However, longitudinal studies and testing the effects of anti-aging interventions are still more challenging in humans than in short-lived animals.

When all of these considerations are taken into account, the dog may rise as an excellent midline solution for the limitations of simple organisms and for the challenges human studies hold (Waters, 2011). Here are some examples, why:

1. Family dogs, on average, age about six to seven times faster than humans. The mean lifespan of companion dogs (purebred and crossbred together) from Europe and Japan were shown to be 12 and 13.7 years, respectively (O’Neill et al., 2013; Inoue et al., 2018), while the mean lifespan of European humans is 77.2, according to a UN report (Anon, 2019) and is around 83 years for Japanese people (Tokudome et al., 2016). Therefore, follow-up studies are much easier in the case of dogs, and have already been performed by several research groups to measure immunological, neuropathological, and metabolomic changes related to canine aging (Su et al., 2005; Greeley et al., 2006; Cotman and Head, 2008).
2. The fact that the mean lifespan of dogs can range from 5.5 to 14.5 years (Michell, 1999; O’Neill et al., 2013; Jimenez, 2016), depending on body size and breed, suggests that dogs, sharing their lives with humans, gained considerable advantages from this alliance by doubling their mean expected lifespan compared to wild wolves (Mech, 2006). This artificially enhances the proportion of individuals with age-related pathologies, which often show strong correspondences with human diseases, and thus can provide opportunities for translational studies.
3. Dogs are prone to develop human-like neurodegenerative disorders and are susceptible to age-related cognitive abnormalities. Almost one third of 11–12-year-old dogs and 70% of 15–16-year-old dogs were reported to show cognitive disturbances with symptoms corresponding to human senile dementia: spatial disorientation, social behavior disorders (e.g., problems with recognizing family members), repetitive (stereotype) behavior, apathy, increased irritability, sleep–wake cycle disruption, incontinence, and reduced ability to accomplish tasks (Neilson et al., 2001). Together, these symptoms constitute a typical, age-related, progressive pathological decline in dogs’ mental abilities, which is usually referred to as “Canine Cognitive Dysfunction Syndrome” (CCD) (Cummings et al., 1996; Landsberg et al., 2012). To this day, a vast amount of literature has accumulated about CCD (Szabó et al., 2016; Chapagain et al., 2018), yet there is weak knowledge about the genetic factors influencing it. Importantly, cognitive decline in dogs was associated with β-amyloid accumulation in the prefrontal cortex, noradrenergic neuron loss in the locus coeruleus (Insua et al., 2010), and, lately, with the formation of tau tangles (Schmidt et al., 2015; Smolek et al., 2016), which can all be seen in humans in early stages of neurodegenerative diseases.
4. Dogs also correspond very well to humans in several metabolic and physiological features, some of which are consequences of domestication (Axelsson et al., 2013). These features have already been thoroughly described in laboratory dogs, as traditional test animals of the pharmacological industry. Therefore, the intestinal absorption profiles of many drugs and supplements are actually known to be very similar in dogs and humans (Roudebush et al., 2005).
5. Several studies from the last two decades (for a review on the history of dog behavioral research, see Feuerbacher and Wynne, 2011) have supported the notion that dogs possess cognitive abilities that are similar to human social skills in communication and learning (Topál et al., 2009; Bensky et al., 2013; Miklósi, 2014). Also, they have a prolonged postnatal period with high sensitivity for human contact and usually live in a close proximity with people, which makes them able to easily interpret many human actions (Miklósi and Kubinyi, 2016). Therefore, dogs can participate in special experimental protocols, which would not be possible with less trainable and sociable species.
6. Dogs share more ancestral genomic sequence with humans than rodents do (Lindblad-Toh et al., 2005), and linkage disequilibrium regions can be extensive within dog breeds, making it easier to pinpoint phenotype–marker associations, which can be later narrowed down by interbreed investigations. This provides particular prospects for GWAS (Boyko, 2011; Vaysse et al., 2011; Schoenebeck and Ostrander, 2014; Hayward et al., 2016).
7. Family dogs are plentiful and easily available at very little cost, so large datasets can be collected *via* the help of dog owners and veterinarians under citizen science approaches (Hecht and Rice, 2015; Stewart et al., 2015).

These points suggest that family dogs can become valuable models to study complex human traits like aging. However, researchers have to face some obstacles and limitations as well, which have to be addressed properly.

1. One of these limitations is the still deficient knowledge about the exact functions that conserved genetic pathways play in canine aging. On the one hand, this may seem to be a minor question, as all fundamental cellular senescence mechanisms were reported to be conserved. On the other hand, divergences may occur in each species regarding some of these mechanisms, as for example, both the telomere biology of flies and the somatic telomerase expression of mice were reported to show marked differences from humans (Kipling and Cooke, 1990; Levis et al., 1993; Prowse and Greidert, 1995). Furthermore, the genes and their functions linked to human age-related neurodegeneration may be fundamentally different from their homologs found in model organisms, or even missing from other species (Bitar and Barry, 2017). Consequently, in an ideal setting, each genetic pathway should be evaluated in each species intended for translational studies before further efforts are put into costly and time-consuming investigations.
2. The variable living environment of family dogs has been discussed as a potential advantage over laboratory dogs; however, it also brings serious challenges to optimal study design. Integrative and cooperative approaches based on large datasets could help to overcome this limitation. Large-scale retrospective studies, which were based on veterinary databases, have already led to important findings regarding the differences in life expectancies between various breeds (Proschowsky et al., 2003; O’Neill et al., 2013; Inoue et al., 2018), or between lean and obese dogs (Salt et al., 2018). In this regard, citizen science approaches can have promising prospects in family dog research (Hecht and Rice, 2015; Stewart et al., 2015), as it was already indicated by a few examples (Hecht and Rice, 2015; Stewart et al., 2015; Ilska et al., 2017). Also, if studies need to involve pathological, histological, and molecular data about dogs that suffered from CCD, citizen science approaches must be expanded to involve a wider range of professionals, including veterinarians. Such interdisciplinary studies may become especially important in cases where family dogs are used as preclinical models to test the anti-aging effects of drugs (Kaeberlein et al., 2016).
3. Until now few studies involved family dogs in cellular and molecular level investigations, as this may require invasive methods or even the sacrifice of animals. Apparently, such approaches are not applicable in the case of family dogs, which, however, represent the valuable genetic and behavioral variability of the species. These issues were encountered by researchers, who aimed to study brains of non-laboratory dogs, and found it difficult to collect both behavioral and molecular data (which required medically advised euthanasia of the animals) from the same individuals within the time-frame of the study (Ghi et al., 2009). In this regard, the establishment and long-term maintenance of databanks and biobanks that collect behavioral, lifestyle, medical data, and biological materials from family dogs, by providing the opportunity for owners to donate their dogs’ bodies for research purposes under appropriate ethical considerations, would be advantageous for canine genomics and aging research.
4. Similarly to invasive methods, genetic manipulations may seem less applicable in dogs than in experimental model organisms. Nevertheless, some groups have already applied targeted genetic manipulations in laboratory dogs to create better models of certain medical conditions (Zou et al., 2015). More importantly, therapeutic applications of gene editing have recently been applied on pet dogs suffering from Duchenne muscular atrophy, with promising results (Amoasii et al., 2018). Hence, it is likely that this line of canine genetics and medical research will continue to unfold its potentials.
5. Currently, methods, by which cognitive aging can be effectively assessed in dogs, are limited. Effective phenotypical categorization of canine age-related pathologies, including CCD, will be crucial for studies, which intend to assess the effects of anti-aging interventions on dogs.

## The Hallmarks of Aging in Dogs

**Figure 1:**
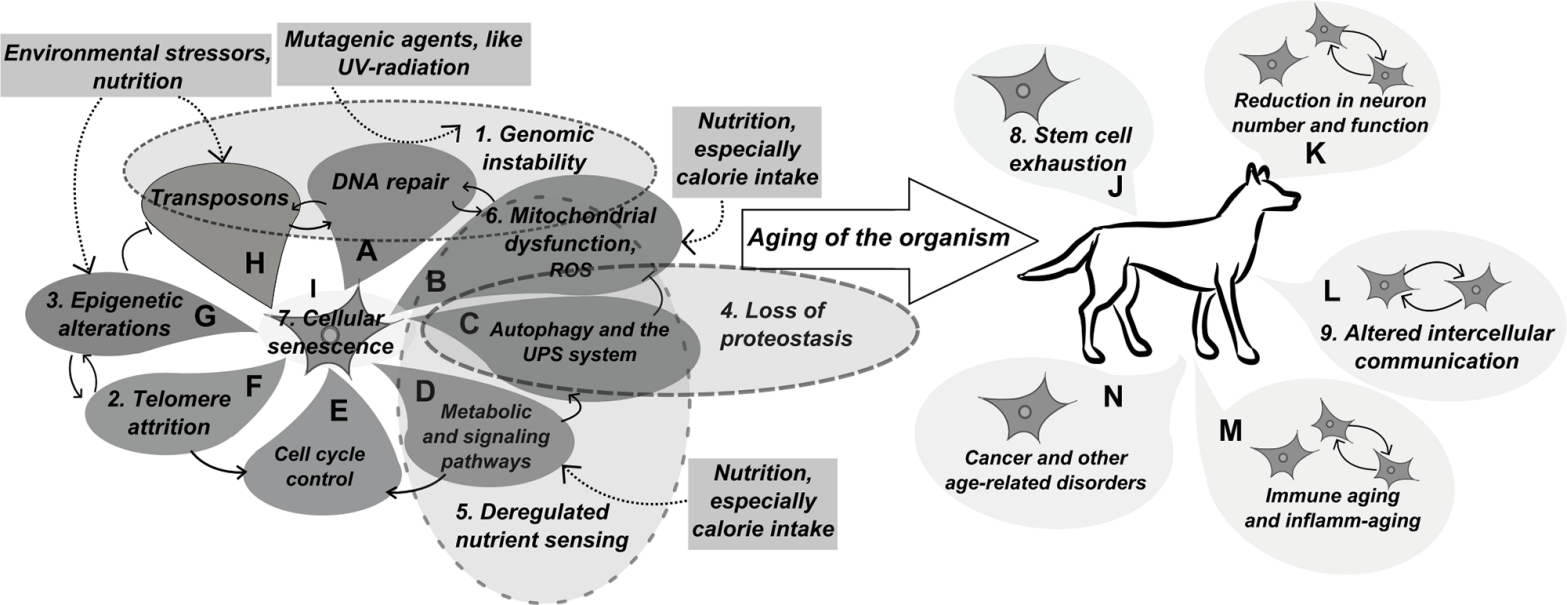
Main mechanisms of aging.The figure depicts the mechanism that underlie cellular aging and, consequently, aging of a multicellular organism. The main mechanisms defined as hallmarks of aging by López-Otín et al. in 2013 are numbered according to their order in the main text. **(A)** The DNA repair machinery provides the first line of defence against mutagenic agents and also interacts with mobile DNA elements through repairing double strand breaks induced by transposons. Impaired function of this machinery can be a main contributor to *Genomic instability* (1). **(B)** Reactive oxygen species produced by mitochondrial respiration represent an inner source for DNA lesions, thus interact with the DNA repair machinery: proper functionality of DNA repair enzymes is required to protect cells from oxidative damage, however elevation in the level of oxidative stress may overburden the repair machinery. Age-related *Mitochondrial dysfunction* (6) can lead to increased oxidative and apoptotic burden. In addition, increased oxidative stress can also affect proteostasis. **(C)** Macroautophagy is a protective mechanism against malfunctioning mitochondria that might cause oxidative stress, while together with other clearance mechanisms it also functions to remove misfolded proteins and protein aggregates. It also functions as a mechanism for programmed cell death (indicated with dashed line between autophagy and cell cycle control). Together with the ubiquitin-proteasome system (not indicated separately on the figure) its malfunction may lead to *Loss of proteostasis* (4) in cells. **(D)** The activity of the autophagy machinery is strictly regulated by different signalling pathways, many of which function in metabolite sensing and cell growth control. Dysruption in these signaling pathways is summarized as *Deregeulated nutrient sensing* (5) by López-Otín et al (2013). **(E)** Cell cycle control is a main determinant of *Cellular senescence* (7). Also, on a multicellular level, dysregulation of cell cycle control may decrease lifespan by initiating tumour formation. **(F)** In many species telomeres function as a measuring mechanisms to limit the number of potential cell cycles. When telomere length reaches a critical shortness, it will activate cell cycle control mechanisms to render the cell into a senescent state. The telomerase enzyme was also shown to interact with global genomic chromatin maintenance. In general, *Telomere attrition* (2) is a main hallmark of aging in most mammalian species. **(G)** Epigenetic regulation involves two main mechanisms, the methylation of CpG islands and modification of the chromatin structure through histone proteins. Chromatin structure at telomeres is important for telomere maintenance and the repression of retroelements by CpG methylation may prevent DNA damage caused by transposon mobilisation. *Epigenetic alterations* (3) are also linked to aging in both humans and model organisms. **(H)** Derepression of mobile DNA elements, primarily of retroelements in mammalian genomes, may result in an increased frequency of double strand breaks and insertion mutagenesis leading to increased *Genomic instability* (1). **(I)** Altogether, the molecular mechanisms of aging eventually result in *Cellular senescence* (7). **(J)** Cellular senescence will lead to reduced function of tissues in a multicellular organism. *Depletion of tissue renewing stem cells* (8) is also a main hallmark of organismal aging and is a result of cellular senescence and increased activation / differentiation of dormant stem cells in certain tissues. **(K)** Loss of stem cells and cellular senescene will lead to functional decline in the central nervous system. **(L)** *Altered intercellular communication* (9) is also considered a main hallmark of aging in multicellular organisms, as coordinated activity of cells is essential for proper tissue functionality. **(M)** The immune system can be affected considerably by altered intercellular communication, and this could also lead to increased inflammation in the body, called inflamm-aging. The elevated levels of inflammation may also result from the increasing number of senescent / apoptotic cells. **(N)** Together with intracellular changes, mainly loss of genomic integrity, disrupted proteostasis and signalling pathways, the imbalanced function of the immune system will contribute to the occurrence of age-related diseases.

Most aging-related genes are components of essential metabolic and signaling pathways (**Figure 1**), like the ones regulating autophagic activity. Other genes make contribution to cellular processes that affect genomic integrity, either in a protective role (DNA repair mechanisms) or in a destructive manner (oxidative stress, transposons). Some genes may affect aging in a somewhat programmed manner, either through epigenetic modulations or by the altered maintenance of telomeres. Because of this large number of involved genetic—and environmental—factors, establishment of a conceptual framework that can systematically comprise all of them would be a first step to provide better insight into the aging process in its entirety. In this regard, recently nine main factors have been designated as fundamental hallmarks that contribute to the aging of animals, with a main focus on mammals (López-Otín et al., 2013). Each hallmark had to meet three criteria: they must affect longevity and healthspan either in a negative or positive manner and have to show age-related changes in measurable parameters. Thus, the following phenomena were defined as main contributors to mammalian aging (**Figure 1**): 1) genomic instability; 2) telomere attrition; 3) epigenetic alterations; 4) disruption of proteostasis; 5) deregulation of nutrient sensing; 6) mitochondrial dysfunction; 7) cellular senescence; 8) stem cell exhaustion; and 9) altered intercellular communication. Although some of these could not perfectly fit all of the criteria, they still make an effective framework to work with. Providing a systematic overview of the genetic pathways involved in the aging of dogs is also of high relevance, as it can help defining directions in canine aging research to support the progression of the species into an effective translational model. For example, in the case of the *apolipoprotein E* (*APOE*) gene, which has polymorphisms strongly associated with average lifespan and Alzheimer’s risk in humans (Nebel et al., 2011; Broer et al., 2015), the translational relevance of the canine homolog is debatable, because *APOE*’s sequence was reported to have low conservation between the two species (Sarasa et al., 2010). Nevertheless, the function of the expressed protein may still be conserved. As *APOE* variants are major risk factors of human dementia, clarifying this question would be an important step to ensure clinical translatability of canine CCD research. In general, exploring more details about genetic pathways and gene variants involved in canine aging and age-related pathologies should be a major consideration of researchers who utilize dogs in aging research.

### Genomic Instability

As organisms age, various forms of damage may accumulate in their genomes, leading to mutations, chromosomal rearrangements, and aneuploidy (Faggioli et al., 2012; Forsberg et al., 2012; Moskalev et al., 2013). Increased mutational burden in somatic cells eventually hinders cellular function and leads to terminal cellular senescence or apoptosis. In cases when cells escape death/senescence inducing processes, malignant transformations can occur as a consequence of genomic damage. Therefore, various protective mechanisms have evolved to prevent or correct DNA damage. Genomic instability arises when the occurrence of deleterious events exceeds the capacity of the DNA damage response system. DNA damaging agents can originate from various extrinsic or intrinsic sources. Intrinsic factors involve oxidative damage, telomere attrition, and transposon insertions.

#### The DNA Repair Machinery

**Figure 2:**
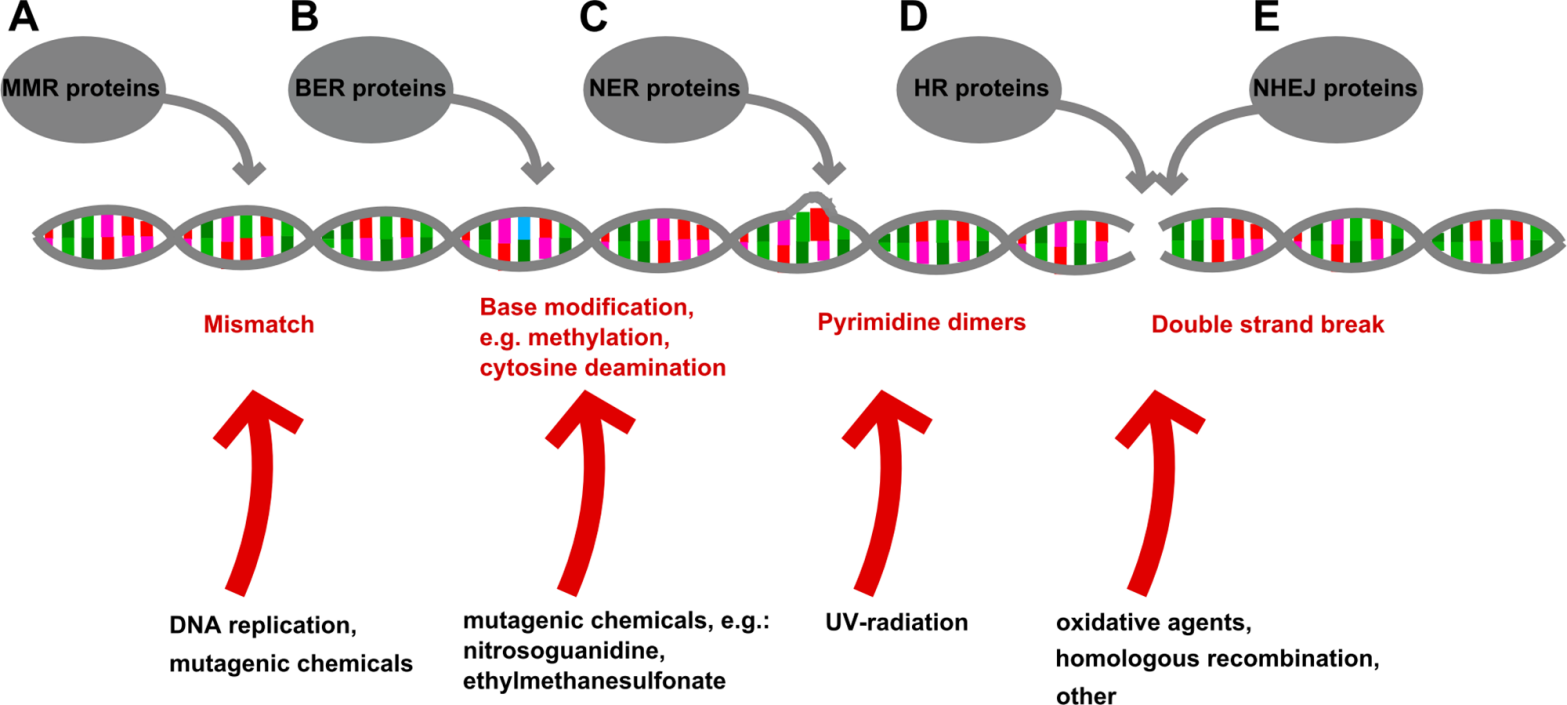
The protective roles of the DNA repair machinery. The DNA repair machinery counteracts the effects of variable DNA damaging processes. In healthy cells, the repair machinery can balance these deleterious effects (represented by red arrows); however, in the case of increased mutagenic burden (e.g., exposure to UV radiation), or when members of the repair machinery are not functioning properly, the balance can be lost and a growing number of DNA lesions may cause the cells to die or turn malignant. **(A)** The function of the Mismatch Repair system (MMR) is coupled to DNA replication where mismatching base pairs can be formed spontaneously and are being identified and repaired by MMR proteins. **(B)** The Base Excision Repair (BER) system can detect damaged/chemically modified bases in the DNA helix, and remove them, resulting in an apurinated site, which will induce endonucleases to cut back the DNA strand. The single strand break is repaired by a DNA polymerase based on the sequence of the complementary strand, and the newly synthesized sequence is ligated to the original DNA strand by a ligase enzyme. **(C)** Mutations that disrupt the normal topology of the DNA double helix, like the UV-light induced formation of pyrimidine dimers, are corrected by the nucleotide excision repair (NER) system. This machinery recognizes aberrant DNA structure caused by chemically modified nucleotides and removes these nucleotides resulting in a single strand break, which will be filled in by DNA polymerase and ligase enzymes. **(D**, **E)** The most destructive form of DNA damage is double strand break (DSB), which could trigger an immediate apoptotic response if it fails to be repaired. Two distinct mechanisms are used by cells to repair DSB: one is homologous recombination (HR) and the other is non-homologous end joining (NHEJ). HR is a fundamental process also linked to meiosis in eukaryotic cells, and it provides a possibility to recover the damaged DNA strand in full length, by using a homologous DNA helix (e.g., the sister chromatid) as template. In contrast, NHEJ may link ends of double stranded DNA together randomly, which could lead to loss of sequences around the breakpoint. All types of DNA repair are indispensable for normal cellular and organismal function.

The DNA repair machinery involves divergent pathways, each aimed to correct certain forms of DNA damage (**Figure 2**). These protective mechanisms have been in the focus of cancer and aging research for a long time (Zimmermann, 1971; Lombard et al., 2005; Cho and Suh, 2014). Defects in DNA repair genes, like the *Bloom syndrome RecQ like helicase* (*BLM*) and the *Werner syndrome RecQ like helicase* (*WRN*), can lead to severe illnesses, called progeria syndromes in humans, which are characterized by premature aging and other symptoms, including cognitive disabilities and a higher rate of tumorigenesis (Ellis et al., 1995; Yu et al., 1996; Martin, 2005; Arora et al., 2014). Mutations in other DNA repair genes were also reported to increase cancer risk (Jeggo et al., 2016) and thus lead to a reduction in expected lifespan. More importantly, polymorphisms in several genes of the DNA damage response machinery have been actually linked to longevity in humans (Cho and Suh, 2014). Intriguingly, no canine progeria syndrome has been documented in scientific literature. On the other hand, several studies that investigated various forms of canine cancer revealed alterations in the DNA repair machinery, which corresponded to findings in human cancers. For example, a reduced DNA damage response capacity was observed in lymphomas of Golden retriever dogs (Thamm et al., 2013), and a lower expression of the *ATM serine/threonine kinase* (*ATM*) gene was found in canine mammary tumors (Raposo-Ferreira et al., 2016). Genetic variations in the *breast cancer 1* (*BRCA1*) and *tumor protein p53* (*TP53*) genes and the *MTAP-CDKN2A* locus were also linked to various forms of cancer in dogs (Kirpensteijn et al., 2008; Rivera et al., 2009; Shearin et al., 2012). While these findings clearly promote the dog as a natural model of human cancers, it is still unclear how exactly variations in DNA repair capacity contribute to the expected lifespan of dogs. A more detailed discussion of DNA repair in dogs can be found in the review of Grosse et al. (2014).

#### Nuclear Architecture

Genomic instability may also rise from altered nuclear architecture. Good examples are the Hutchinson-Gilford and the Néstor-Guillermo progeria syndromes, which were linked to mutations in *lamin* genes responsible for formation of the nuclear lamina (De Sandre-Giovannoli et al., 2003; Eriksson et al., 2003; Cabanillas et al., 2011). In addition, both the accumulation of progerin, which is an aberrant form of lamin A, and the reduced expression of lamin B1 were linked to aging (Freund et al., 2012; Golubtsova et al., 2016; Hilton et al., 2017), and lamins were also shown to regulate DNA damage response (Gonzalez-Suarez et al., 2009). Thus, the canine homologs of *lamins* could be promising targets in aging research. So far, a few studies have investigated them, mainly in regard to their possible role in hereditary diseases, like dilated cardiomyopathy of Doberman pinschers and Newfoundland dogs (Wiersma et al., 2007; Meurs et al., 2008) and elbow dysplasia in Bernese Mountain Dogs (Pfahler and Distl, 2012); however, only in the latter case an association was reported between disease occurrence and the *lamin B1* gene.

#### Oxidative Damage

Oxidative damage in cells mainly results from chemical interactions between cellular constituents and reactive oxygen species (ROS), which chemically act as free radicals, characterized by a high oxidative activity. These agents target macromolecules, such as DNA, lipids (Rubbo et al., 1994), and proteins (Stadtman and Levine, 2006), and thus may make a huge impact on cellular function. The sources of ROS are many: mitochondrial respiration (Liu et al., 2002; Murphy, 2009), ionizing radiation (Riley, 1994), and the activity of specific enzymes, such as the NADPH oxidase (Babior, 2004) and dual oxidase (DUOX) (Edens et al., 2001), are the main examples.

The amount of oxidative DNA lesions has been well documented to increase with age in different species. For example, in rats, Fraga et al. (1990) reported the age-related accumulation of 8-hydroxy-2-deoxyguanosine, which is a typical product of DNA oxidation. Furthermore, several studies confirmed that aged dogs show elevated levels of oxidative damage in their brains, indicated by the accumulation of carbonyl groups (Head et al., 2002; Skoumalova et al., 2003), lipofuscin (Rofina et al., 2004), 4-hydroxynonenal (Papaioannou et al., 2001; Rofina et al., 2004; Hwang et al., 2008), and malondialdehyde (Head et al., 2002) in neural tissue. In addition, as the reduced expression of antioxidant enzymes may also contribute to the increased oxidative burden in cells (Kiatipattanasakul et al., 1996), their role in neural aging and neurodegeneration should also be considered. In humans, for example, a mutation in superoxide dismutase 1 (SOD1), which is a main antioxidant enzyme in cells, has been linked to amyotrophic lateral sclerosis (ALS) (Orrell, 2000). Importantly, a mutation in the canine homolog of *SOD1* was also linked to an ALS like neurodegenerative process, called degenerative myelopathy (DM) (Awano et al., 2009).

Surprisingly, ROS have also been indicated as important and evolutionary conserved signaling molecules, which function in pathways that respond to availability of nutrients, changes in environmental oxygen levels, and exercise (Schieber and Chandel, 2014; Merry and Ristow, 2016). Hydrogen peroxide (H_2_O_2_), for example, plays an important role as signal transducer in the MAPK and Nf-Kβ pathways (Allen and Tresini, 2000) and also serves as activator of peroxiredoxins (Wood et al., 2003), which are crucial for maintaining redox balance of cells. Nitric oxide (NO) has long been indicated to play various physiological roles, with emphasis on the immune and cardiovascular systems (Lundberg et al., 2008). Thus, maintaining optimal levels of oxidative stress in cells could actually be more important for healthy aging than maximizing the neutralization of ROS by antioxidants. In accordance with this, some studies reported controversial effects of oxidative stress in aging and metabolic parameters. For example, it was demonstrated by Ristow et al. (2009) in a human study that supplementation with high doses of extrinsic antioxidants ameliorated the beneficial effects of exercise in volunteers. Furthermore, elevated levels of ROS were reported to either increase lifespan in yeast and worms (Doonan et al., 2008; Van Raamsdonk and Hekimi, 2009; Mesquita et al., 2010) while having no effect on mortality in mice (Van Remmen et al., 2003; Zhang et al., 2009). Also, the lifespan extension of worms promoted by reduced glucose availability was found to be accompanied by elevated levels of ROS in cells (Schulz et al., 2007). Such findings led to the reconsideration of the role oxidative stress plays in cellular senescence and resulted in a more refined view (López-Otín et al., 2013; Shadel and Horvath, 2015). In this regard, it is a question yet to be addressed, how lifelong antioxidant supplementation, often provided by high-quality commercial foods, may affect the healthspan of dogs.

#### Transposable Elements

**Figure 3:**
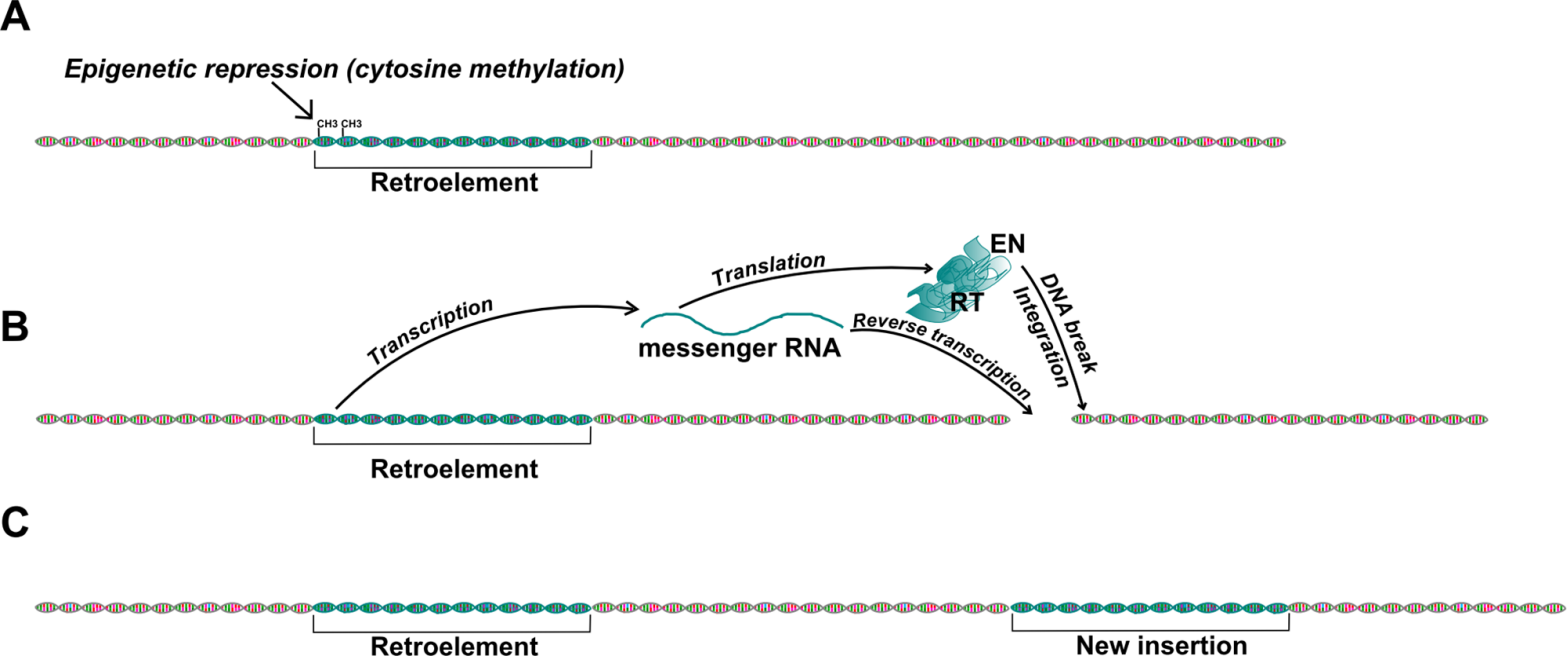
Mobilization of retroelements in the genome. This picture shows the basic mechanism of retrotransposon mobilization. **(A)** Normally, activity of functional retroelements, like LINE-1, is repressed in somatic cells by methylation of CpG islands in their promoter regions. **(B)** Demethylation of the transposon promoter may result in transcriptional activation. The transcribed mRNA encodes the proteins necessary for retrotransposition, the Integrase (Int) and Reverse Transcriptase (RT), and also serves as template for reverse transcription. The reverse transcribed transposon DNA will be integrated into the genome by the Int protein, which first induces a double strand break. **(C)** The retroelement has copied itself into a new genomic region.

The mobilization of endogenous transposable elements, called transposons, has recently gained attention as an intrinsic contributor to cellular senescence (Gorbunova et al., 2014). Transposons are present in the genomes of all organisms, from bacteria to mammals, and possess the ability to change their position in or between chromosomes. They can be categorized into two groups. Retrotransposons move by a replicative “copy and paste” mechanism, increasing in numbers in their host genome (**Figure 3**), while DNA transposons mainly follow a “cut and paste” mechanism, leaving only a short footprint behind (Wicker et al., 2007; Mandal and Kazazian, 2008). Since the human genome project revealed that around 55% of the human genome is composed of remains of transposable elements, mainly of retrotransposons, an increased attention has been paid to their role in genome evolution (Kazazian, 2004), especially in the formation of gene regulatory networks (Sundaram et al., 2014). While all DNA transposons have lost their mobility in the course of human evolution (Pace and Feschotte, 2007), several retroelements found in our genome are still active and can cause insertional mutations (Hancks and Kazazian, 2016). Human retroelements can be categorized into three groups: the *LTR* (*Long Terminal Repeat*) elements, the *LINE* (*Long Interspersed Nuclear Element*) transposons, and the *SINE* (*Short Interspersed Nuclear Element*) transposons (Mandal and Kazazian, 2008). Similar types of retroelements can be found in the dog genome; however, transposon-derived sequences make up only 34% of it (Lindblad-Toh et al., 2005). Importantly, active *LINE* and *SINE* elements are present in both species.

Active retroelements have been found responsible for several hereditary diseases in dogs by causing insertional mutations. For example, a *LINE-1* (*L1*) insertion in the gene of Factor IX was shown to segregate with mild hemophilia in German Wirehaired Pointers (Brooks et al., 2003), while a similar insertion in the *dystrophin* gene leads to Duchenne-like muscular dystrophy in Pembroke Welsh Corgi dogs (Smith et al., 2011). *SINE* elements were also shown to cause several inherited diseases, like recessive centronuclear myopathy in Labrador Retrievers (Tiret et al., 2005) and early canine retinal degeneration, which was linked to the *serine/threonine kinase 38 like* (*STK38L*) gene in Norwegian Elkhound–Beagle outcrosses by linkage mapping (Goldstein et al., 2010). A form of progressive retinal atrophy (PRA) in Tibetan Spaniels and Tibetan Terriers was also associated with a *SINE* insertion, but in the *family with sequence similarity 161 member A* (*FAM161A*) gene (Downs et al., 2014). Bandera’s neonatal ataxia in Coton de Tulear dogs was shown to be caused by the disruption of the *glutamate metabotropic receptor 1* (*GRM1*) gene by recent retrotransposon mobilization, as the insertion was not found in other breeds (Zeng et al., 2011). Interestingly, several examples of non-disease causing insertional mutations are known, which alter morphology (Parker et al., 2009; Marchant et al., 2017) or coat color (Clark et al., 2006; Dreger and Schmutz, 2011) and thus have become selection criteria in many breeds. Beyond these examples, where the integration event can be revealed by a phenotypic effect or disease, the mobilization of retroelements seems common in dogs, as analyses of individual dog genomes showed that approximately half of annotated dog genes contain a SINEC_Cf type insertion in their introns (Wang and Kirkness, 2005). This high activity of retrotransposons in the lineage of domestic dogs can be explained by intense selection pressures that resulted from domestication, breeding strategies, and changing environment (Capy et al., 2000; Chénais et al., 2012). This hypothesis was actually supported by the findings of Koch et al. (2016), who compared the methylation patterns of wolf and dog genomes and found that almost half of the sites potentially relevant in domestication contained a *LINE* or *SINE* insertion.

Beyond the germ-line mutations discussed so far, a vast body of evidence indicates that retroelements can mobilize in somatic cells (Collier and Largaespada, 2007; Hunter et al., 2015) although this is strictly controlled by specific non-coding small RNAs and epigenetic regulation, including hypermethylation and transcriptional repression (Levin and Moran, 2011; Pizarro and Cristofari, 2016). As the general hypomethylation of the genome has long been documented to be an attribute of aging (Wilson and Jones, 1983; Singhal et al., 1987), more and more researchers have suggested a main role for somatic transposon mobilization in cellular senescence (Murray, 1990; Sturm et al., 2015; Pal and Tyler, 2016). Importantly, this hypothesis was also supported by experimental findings. For example, the artificial downregulation of the yeast *Ty1* element resulted in lower levels of age-related chromosome rearrangements in aged cells (Maxwell et al., 2011). Also, many of the 118 *L1* subfamilies of mice showed an elevated expression with age (De Cecco et al., 2013). In humans the hypomethylation of *LINE-1* and *Alu* (a *SINE* element abundant in the human genome) elements has been linked to cancer susceptibility (Zhu et al., 2011; Luo et al., 2014).

Because the activation of transposable elements can be induced by various environmental stressors as well (Hunter et al., 2015), including heavy metal toxicity (Morales et al., 2015), certain genotoxic agents (Stribinskis and Ramos, 2006), and even nutrition (Waterland and Jirtle, 2003), they represent another possible intracellular interface between the living environment/lifestyle and aging. In this regard, family dogs, which share their environment with their owners, can be valuable models to study how retrotransposons may contribute to aging and mortality under various circumstances. The age-related activity of retroelements has not yet been specifically assessed in dogs. However, in a study that investigated the elevated blood levels of *SINE* sequences in dogs with mammary tumors, it was shown that tumor-affected dogs above 10 years of age had higher levels of circulating *SINE* elements than younger dogs with tumors (Gelaleti et al., 2014).

### Telomere Attrition and Aging

**Figure 4:**
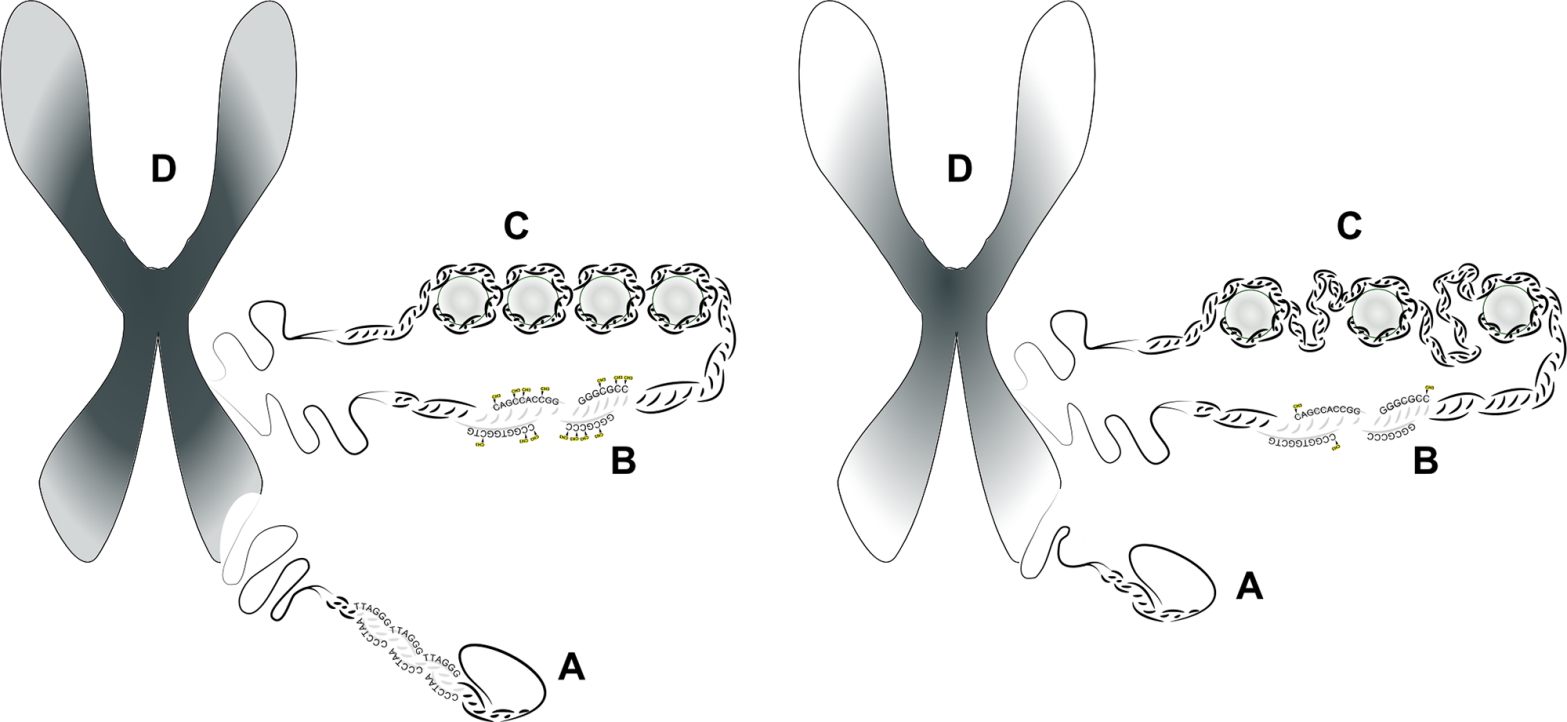
The role of telomeres and epigenetics in chromosomal integrity and aging. The figure illustrates how shortening of telomeres and changes in the epigenetic pattern affect the overall structure of chromosomes. **(A)** Chromosome ends are protected by repetitive sequences called telomeres in most eukaryotic organisms. This telomere sequence, consisting of TTAGGG repeats, shortens with each DNA replication, which eventually triggers cellular senescence. **(B)** Chromatin changes occur on the first level of DNA packaging, when the DNA double strand is coiled up on nucleosomes. Tight coiling on nucleosomes results in a heterochromatic state, when the DNA double helix is not accessible to many proteins, including the transcription machinery. In contrast, reduction in the number of nucleosomes leads to a less coiled and less dense state rendering the DNA more open to transcription. **(C)** DNA methylation at CpG islands cause chemical changes directly in the DNA double helix. Cytosine methylation is usually linked to silencing of transcription. Methylation also interacts with chromatin structure: increased CpG methylation is usually linked to heterochromatic state. **(D)** Changes in chromosomal structure during aging is characterized by a decrease of heterochromatic regions (symbolized by darker color) and an increase of euchromatic regions (symbolized by lighter colors).

In most eukaryotic cells, shortening of the protective sequences at chromosome ends (**Figure 4A**), called telomeres, occurs with each DNA replication. Therefore, telomere shortening has been proposed as a key mechanism of cellular senescence and it also suggested the existence of an aging program in cells (Shampay and Blackburn, 1988; Harley et al., 1990; Hastie et al., 1990). This so called replicative aging limits the number of cell cycles a cell can go through before reaching its Hayflick limit and entering a senescent state (Hayflick, 1976). Furthermore, a recent study supported the conserved role of this aging program on the level of whole organisms by reporting clear correlations between the rate of telomere shortening and the lifespan of different mammalian and bird species (Whittemore et al., 2019). Importantly, telomere shortening is a characteristic only of somatic cells, while in germ line cells, telomere sequences are constantly restored by telomerase enzymes. The limited proliferative potential of somatic cells may seem disadvantageous for an individual, yet it may increase fitness by limiting the growth of malignant cells. In line with this, recent studies have suggested a trade-off between telomere length and cancer occurrence (Zhang et al., 2015; Stone et al., 2016). On the other hand, loss of telomeres can result in end-to-end chromosomal fusions, which might also lead to tumorigenesis (Hastie et al., 1990). These findings indicated that fine-tuning of telomere dynamics in somatic cells might be crucial for healthy aging, at the cost of reducing the maximal lifespan. In fact, polymorphisms in genes associated with telomerase function were shown to be linked with expected lifespan and disease predisposition in human populations (Atzmon et al., 2010; Soerensen et al., 2012b; Codd et al., 2013). Telomere dynamics may also play an influential role in neurodegeneration, as patients with Alzheimer’s showed shorter average telomere length than healthy controls (Forero et al., 2016). Therefore, understanding the links between telomeres and age-related changes on the cellular level, which can lead to pathological processes, is a main goal in aging research. However, in some animals, including common laboratory models, telomerase biology does not entirely correspond to that described in humans. The laboratory mouse, for instance, was shown to exhibit a high variability of telomere length and telomerase expression in adult tissues (Kipling and Cooke, 1990; Prowse and Greidert, 1995; Greenberg et al., 1998; Martín-Rivera et al., 1998), indicating a lesser role of constant telomere attrition as a programmed aging inducer. More importantly, *D. melanogaster* was shown to possess a fundamentally different telomere structure than found in other animals, as chromosome ends of fruit flies are capped by transposon derived sequences (Levis et al., 1993). These facts clearly limit the applicability of these species as models of human telomere function (Wright and Shay, 2000; Smogorzewska and De Lange, 2002). Nevertheless, longevity of mice was still shown to be positively affected by gene therapy induced telomerase expression (de Jesus et al., 2012).

Contrary to mice, dogs were reported to have low or no telomerase expression in normal somatic tissues, a pattern similar to that in humans (Nasir et al., 2001). It was also reported by Yazawa et al. (1999) Yazawa et al. (2001). Furthermore, tumors in dogs often showed high levels of telomerase expression, similarly to human malignancies (Vonderheide et al., 1999; Lamb et al., 2015). Although very little is known about the molecular mechanisms regulating telomere maintenance and cell cycle arrest in dogs, such findings indicate that dogs may also share basic telomere biology with humans. Importantly, telomere length was shown to be variable across different dog breeds and was in correlation with expected lifespan (Fick et al., 2012). Also, telomere length in individual dogs was found to decrease with age (Nasir et al., 2001), similarly as described in humans (Harley et al., 1990; Hastie et al., 1990; Lindsey et al., 1991).

### Epigenetic Alterations

Epigenetics refers to mechanisms that modulate gene expression by determining how the transcription apparatus can access different sections of the genomic DNA. The condensation procedure, which literally packs the DNA double helix into a dense structure, called chromatin, is one of the main mechanisms to provide epigenetic regulatory potentials (**Figure 4C**). The structure of chromatin is determined by histone proteins, which constitute the basic building blocks for DNA condensation, the nucleosomes. The more densely packed heterochromatic state renders the DNA inaccessible for RNA polymerases and thus inhibits gene expression, while genes positioned in euchromatic sites are open to transcription. However, not all of these genes can be transcribed, even if appropriate activating factors are present, as another epigenetic mechanism, the methylation of cytosines at specific GC-rich sites (called CpG islands), may block transcription (**Figure 4B**). This process has an important role in cellular differentiation and probably also acts as genomic “memory,” storing information about the fate of individual cells (Bird, 2002; Halley-Stott and Gurdon, 2013). Abnormal somatic alterations in DNA methylation have been linked to various diseases, including schizophrenia (Hannon et al., 2015; Wockner et al., 2015). Furthermore, changes in chromatin structure and methylation pattern are often found in cancer (Daniel and Tollefsbol, 2015), where the disruption of cellular identity and concurrent dedifferentiation is a common phenomenon.

Interestingly, the genomic methylation pattern is erased and rewritten during spermato- and oogenesis and after fertilization in mammals (Geiman and Muegge, 2009; Seisenberger et al., 2012; Smith et al., 2012). The exact role these mechanisms play in aging, however, is still unknown.

In general, systemic changes in the ratio of heterochromatic and euchromatic regions (**Figure 4D**) and a global hypomethylation of the genome have been shown to accompany aging (Gentilini et al., 2013; Pal and Tyler, 2016). When focusing on specific genomic regions, however, both hypomethylation and hypermethylation should be taken into account (Maegawa et al., 2010). Actually, senescence-related changes in the DNA methylation profile may include both the activation of pro-aging genes and the repression of anti-aging genes, as in the case of *WRN* and *LMNA* (Fraga and Esteller, 2007). The remodeling of chromatin structure, induced by methylation and acetylation of certain histone protein residues, also shows complex age-related patterns (Fraga and Esteller, 2007; Han and Brunet, 2012). Importantly, both chromatin dynamics and DNA methylation were shown to interact with other age-related genetic pathways, like telomere-length control (Blasco, 2007). In turn, the telomerase enzyme was found to affect chromatin structure and DNA repair mechanisms (Masutomi et al., 2005). In addition, the epigenetic pattern is regulated by many factors other than developmental status, like stress, exercise, and diet (Daniel and Tollefsbol, 2015), which therefore can also affect aging through altering the expression of certain genes.

Although age associated changes in chromatin structure and DNA methylation patterns have been reported in several model animals, there can be major differences between species. For example, epigenetic regulation in *C. elegans* seems to be limited to chromatin remodeling by histone modifications, as m5C DNA methylation pattern does not exist in this organism (Bird, 2002), limiting its utilization as a model to study epigenetic changes in aging. Nevertheless, the histone demethylase UTX-1 was shown to regulate aging in worms (Jin et al., 2011).

In dogs, an increasing body of evidence has suggested epigenetic regulation behind species and breed-specific traits (Koch et al., 2016; Banlaki et al., 2017; Cimarelli et al., 2017). Importantly, a recent study demonstrated that changes in methylation status in DNA regions, which were homologous to regions with known age-sensitive methylation patterns in humans, were in strong correlation with chronological age in dogs and wolves (Thompson et al., 2017). This finding supported the applicability of the dog as a model of age-related epigenetic changes, while it also provided a molecular approach to determine the biological age of individual canines.

#### Regulation of Epigenetic Pattern

The regulation and maintenance of the epigenetic pattern are coordinated by various enzymes, which act downstream of metabolic and signaling pathways. Altered functions of these enzymes were shown to have a major impact on health and aging. Most importantly, *sirtuin* genes were among the first shown to affect longevity in yeast (Kaeberlein et al., 1999), *C. elegans* (Tissenbaum and Guarente, 2001), *Drosophila* (Rogina and Helfand, 2004), and mice (Calvanese et al., 2009). Sirtuins exert various enzymatic functions, including histone deacetylation, and thus play a key role in the maintenance of chromatin structure (Longo and Kennedy, 2006; Fraga and Esteller, 2007). They also interact with many signaling and metabolic pathways, and regulate oxidative metabolism, stress response, autophagy, and the maintenance of telomeres (Jia et al., 2012; Kim et al., 2012). In mammals, seven *sirtuins* are known with divergent functions (Guarente, 2011) and at least three of them—*SIRT1*, *SIRT3*, and *SIRT6*—have been implicated to modulate aging. Importantly, polymorphisms in *sirtuin* genes have been actually linked to human longevity (Kim et al., 2012; Albani et al., 2014).

Sirtuins and other histone-modifying enzymes, together with DNA methyltransferases, have been barely studied in dogs so far. However, as the sequence and function of *sirtuin* genes show a highly conserved nature (Greiss and Gartner, 2009; Gaur et al., 2017), they are likely to play similar roles in the aging of dogs as in other species. In fact, altered expression of *sirtuin* genes, mainly that of *SIRT1*, have been implicated in canine tumors (Marfe et al., 2012), similarly as in humans (Brooks and Gu, 2009). Several sirtuin-targeting drugs have been proposed as promising pharmacological interventions to fight disease and aging (Dai et al., 2018); therefore, they are likely to be utilized in the future as anti-aging therapeutics and may be applied in dogs as well. In this regard, some compounds that interact with histone-regulating enzymes have already been tested in dogs for various reasons. For example, the histone deacetylase inhibitors AR-42 and panobiostat showed promising results in dog cell line models of prostate cancer and B-cell lymphoma, respectively (Elshafae et al., 2017; Dias et al., 2018). More importantly, resveratrol, which has sirtuin-activating effect (Gertz et al., 2012), was reported to positively affect the immune function of healthy pet dogs (Mathew et al., 2018) and it also effectively inhibited the growth of canine hemangiosarcoma *in vitro* (Alderete et al., 2017). Actually, resveratrol is one of the most comprehensively studied naturally occurring compounds with suggested beneficial effects on health and aging. It was shown to activate SIRT1 and improve mitochondrial function in mice (Lagouge et al., 2006) and to reverse age-related cognitive decline in learning and memory in rats (Gocmez et al., 2016). However, its longevity benefits are still dubious. For example, it only increased the relative survival of mice when the animals received a high calorie diet (Baur et al., 2006). Longitudinal follow-up studies on family dogs may help to clarify this question, as these animals represent a naturally variable population regarding diet and genetic background.

#### Age-Related Changes in Gene Expression

Alterations in the epigenetic pattern, together with the availability of transcription factors and activation of signaling pathways, can influence the whole expressed mRNA content (the transcriptome) in cells. Not surprisingly, altered gene expression patterns were shown to correlate with aging in mice, humans, and dogs (Lee et al., 1999; Lee et al., 2000; Lu et al., 2004; Zahn et al., 2007; Swanson et al., 2009). Comparisons between species-specific expression profiles have already been implicated as powerful tools to identify evolutionary conserved regulatory pathways (de Magalhães et al., 2009). In this regard, further gene expression data from dogs, especially from individuals with CCD, may also help researchers to pinpoint the shared molecular pathways of human and canine neurodegeneration.

The expression of some microRNAs (miRNAs), which are small non-coding RNAs with important regulatory functions, was also shown to correlate with aging in humans and mice (Somel et al., 2010; Drummond et al., 2011; Inukai et al., 2012; Zhang et al., 2012). Furthermore, age-associated miRNAs—named as gero-miRNAs—were identified in various organisms and were shown to target mRNAs associated with longevity pathways (Gonskikh and Polacek, 2017). Thus, characterization of gero-miRNAs would be a crucial step in dog aging research to further support the role of the dog as a translational model of human aging. Efforts have already been made to provide a detailed annotation of canine miRNAs (Penso-Dolfin et al., 2016), including the establishment of a miRNA tissue atlas in Beagle dogs (Koenig et al., 2016).

### Disruption of Proteostasis

Proteins represent the key functional components of cells. The totality of all protein types expressed simultaneously in a cell is called the proteome. Proteome integrity is indispensable for the optimal functionality of cells; therefore, several mechanisms have evolved to maintain its homeostasis—called proteostasis. Impairments in proteostasis can lead to cellular senescence and even severe diseases, called proteinopathies, which mainly affect the CNS and are caused by the excessive accumulation and aggregation of misfolded proteins (Pievani et al., 2014). Loss of proteostasis is hypothesized to be a general attribute of aging cells across different taxa (Koga et al., 2011). For example, it was reported to be an early sign of aging in worms (Ben-Zvi et al., 2009) and to be a characteristic change during both premature and normal aging in mice (Wilson et al., 2015).

Proteostasis is maintained by the orchestrated function of mechanisms, which provide protein quality control, support the folding of synthesized proteins, protect them from various stressors, and eventually remove aberrant or senescent proteins from the cell. The folding and stability of proteins are mainly supervised by so called chaperone proteins, while the efficient removal of unnecessary, damaged, or senescent proteins is handled by two machineries: the ubiquitin-proteasome system (UPS) and the autophagy-lysosome pathway.

#### Chaperones and Protein Quality Control

Chaperone proteins play an important role in the post-translational maturation of nascent proteins by facilitating their folding. They also function as protectors of mature proteins under various stressful conditions, by helping to maintain their natural conformation and by preventing aggregation. Actually, the first identified chaperones were named heat shock proteins (Hsp), because their expression was induced by elevated temperatures. Importantly, many of these stress responsive chaperones were reported to show reduced expression with aging (Calderwood et al., 2009), and genetic manipulations that affected the expression of certain heat shock proteins resulted in altered aging phenotypes in model organisms. Overexpression or upregulation of Hsp-s was shown to extend lifespan, together with providing increased stress resistance, in both worms and flies (Walker and Lithgow, 2003; Morrow et al., 2004; Chiang et al., 2012), while reduced chaperone function caused accelerated aging in mice (Min et al., 2008).

In humans, chaperones, together with other proteostasis maintenance mechanisms, were suggested to play important roles in neurodegenerative diseases (Morimoto, 2008). This role, however, may not be entirely protective, as some Hsp-s were actually indicated to augment propagation of malformed proteins in proteinopathies (Dickey et al., 2007; Luo et al., 2007).

In dogs, the few studies that investigated chaperone proteins in relation to aging reported similar age-related changes as in humans. For example, blood levels of the Hsp70 chaperone were shown to decrease with age in dogs (Alexander et al., 2018), similarly to what had been previously reported in humans (Deguchi et al., 1988). Interestingly, a research group that investigated the hippocampi of donated pet dogs from various breeds (Ghi et al., 2009) reported an age-related increase in Hsp90 levels. This finding could indicate both a compensatory response to the accumulation of damaged proteins and a more direct link between Hsp90 and age-related neural decline in dogs, similarly as it was suggested in humans, where Hsp90 was implicated as a factor that may actually drive spreading of taupathy (Dickey et al., 2007; Luo et al., 2007). Based on these possible similarities between canine and human chaperone functions in the brain, dogs can be suitable to test various pharmacological interventions and small molecular chaperones (Calamini et al., 2012), which modify or complement chaperone activity to support proteostasis and reduce neurodegenerative pathologies. Such interventions have already been successfully tested in rodents (Gehrig et al., 2012).

#### The Ubiquitin-Proteasome System

The UPS is responsible for the selective removal of misfolded and senescent proteins in cells. Mutations in genes that encode subunits of the proteasome and proteins responsible for proteasomal targeting can lead to accumulation of aberrant proteins, and have been actually linked to several types of neurodegenerative diseases, including Alzheimer’s disease and Parkinson’s disease (Zheng et al., 2016). Especially in the case of AD, causative links were described between disturbances in the UPS and progression of the disease (de Vrij et al., 2004; Liu et al., 2014).

Importantly, the UPS was also linked to longevity in model organisms. In flies, loss-of-function mutations in the ubiquitin activating enzyme E1 were shown to reduce lifespan and cause disturbances in motor function (Liu and Pfleger, 2013); meanwhile, extended lifespan in worms was associated with increased expression of a proteasome subunit (Vilchez et al., 2012).

In dogs, an increased density of ubiquitinated bodies was reported to be present in the brains of aged individuals (Ferrer et al., 1993; Borràs et al., 1999), and further signs of impaired proteostasis were also indicated (reviewed by Romanucci and Della Salda, 2015). The same group that reported age-related increase in the Hsp90 chaperone in dog brains also found incongruent changes in the abundance of various proteasomal proteins, suggesting complex impairments and compensatory mechanisms in the regulation of the UPS in aged dogs (Ghi et al., 2009).

Interestingly, a homozygous lethal mutation in the proteasome β2 subunit was reported as the possible causative variant behind the unique harlequin coat color of Great Dane dogs (Clark et al., 2011). Further studies may shed light on the possible health or longevity effects of this mutation.

Importantly, as proteasome activation by pharmacological agents has been proposed as a promising approach to delay aging and the development of age-related diseases (Chondrogianni et al., 2015), dogs may provide an appropriate large animal model for pre-clinical testing of these interventions, especially in the case of brain pathologies.

#### Autophagy

**Figure 5:**
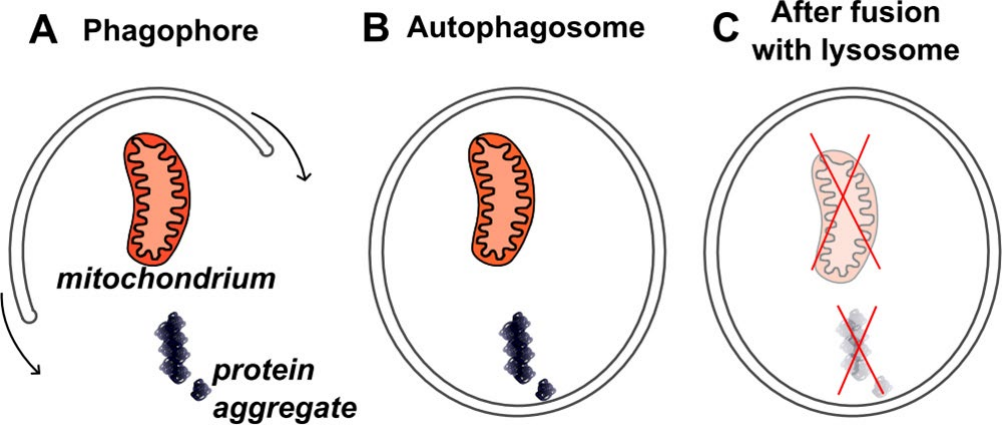
Macroautophagy. This figure depicts macroautophagy, which is the only mechanism in cells able to remove aberrant mitochondria and large protein aggregates. **(A)** First, formation of a double membrane structure, the phagophore, is initiated around the target. **(B)** The expansion of the double membrane structure around the target will eventually form a vesicle, called autophagosome. **(C)** When the autophagosome fuses with a lysosome, degradation of the autophagosome’s content can take place and resulting molecular compounds can be recycled thereafter.

While the UPS eliminates individual proteins or small aggregates tagged by ubiquitin, the autophagic machinery is capable of targeting greater amounts of cellular content for lysosomal degradation, including mitochondria and large protein aggregates. In fact, autophagy is a fundamental mechanism in eukaryotic cells and was often found indispensable for the ontogenesis of multicellular organisms, including the embryonic development of mice (Cecconi and Levine, 2008) and the metamorphosis of flies (Juhász et al., 2003). Three main types of autophagy have been described in the literature. In the case of macroautophagy, targeted cytoplasmic constituents get isolated by double membrane vesicles, called autophagosomes. These then fuse with lysosomes, leading to the degradation of their content into small molecular components (**Figure 5**), which can be recycled thereafter (Klionsky, 2005). The other two types, microautophagy and chaperone-mediated autophagy, utilize different targeting mechanisms and may be less capable for bulk degradation of intracellular content. Therefore, the name autophagy usually refers to macroautophagy in the literature. In general, all types of autophagy play a crucial role in cellular metabolism (Rabinowitz and White, 2010); pathogen resistance (Levine, 2005; Deretic, 2006); inflammation (Levine et al., 2011); and cleansing of macromolecular debris, like protein aggregates seen in Alzheimer’s disease (Rubinsztein, 2006; Mizushima et al., 2008; Nixon and Yang, 2011) and in programmed cell death (Tsujimoto and Shimizu, 2005; Ouyang et al., 2012). Impairments in autophagy were linked to several disease phenotypes in model organisms, as well as in dogs (listed below) and humans (Levine and Kroemer, 2008). Importantly, reduced autophagic activity in the adult brain was shown to promote neurodegeneration in mice (Hara et al., 2006; Komatsu et al., 2006).

Based on these findings, it is not surprising that autophagy has been proposed as a major factor in aging regulation. In *C. elegans*, loss-of-function mutations in autophagy genes shortened lifespan, while disruption of signaling pathways that downregulate autophagy led to a significant increase in expected lifespan (Hars et al., 2007; Tóth et al., 2008). Similar findings were reported from yeast, flies, and mice (Juhász et al., 2007; Simonsen et al., 2008; Eisenberg et al., 2009; Pyo et al., 2013), although with a less pronounced lifespan extension in the latter. Importantly, the longevity effect of caloric restriction (CR)—which is discussed in the **Supplementary Section** “Beyond Genetics”—was shown to be dependent on the proper functioning of autophagy (Jia and Levine, 2007). Chaperon-mediated autophagy was also reported to directly affect cellular senescence through the selective elimination of soluble proteins (Cuervo and Dice, 2000; Massey et al., 2006; Zhang and Cuervo, 2008). In the livers of aged mice, but not of young animals, impaired function of chaperone-mediated autophagy resulted in increased loss of proteostasis (Schneider et al., 2015).

Surprisingly, cohort studies have reported little or no association between autophagy linked genes and longevity in humans, implicating that the effects of mutations, which alter autophagic activity, are less pronounced, or that such mutations are not common in people. Nevertheless, the role autophagy has in neurodegenerative processes is indisputable in humans (Jiang and Mizushima, 2014). For example, mutations in the *WD repeat domain 45* (*WDR45*) gene, which functions in formation of the double membrane structures (“phagophores”), were shown to cause static encephalopathy of childhood with neurodegeneration in adulthood (SENDA). Also, both Alzheimer’s and Parkinson’s diseases were characterized by accumulation of autophagic vacuoles, indicating a disruption in their turnover (Nixon et al., 2005; Nixon and Yang, 2011). Consonantly, loss-of-function mutations in the *Parkinson’s disease associated protein DJ-1* gene were linked to reduced basal levels of autophagy (Krebiehl et al., 2010). On the other hand, enhanced levels of autophagy have been linked to neuron loss in ALS (Sasaki, 2011; Chen et al., 2012), marking it as a possible driver of neurodegeneration in this case. Such controversial findings may result from the complex roles autophagy plays in cellular homeostasis, stress resistance, and also in programmed cell death (White and DiPaola, 2009; Tung et al., 2012), calling for further research to clarify its contribution to different types of neurodegeneration. In this regard, the dog could serve as a model more closely related to human physiology than rodents. Some canine hereditary diseases have already been linked to mutations in autophagy genes and many of these diseases have human homologs. For example, a polymorphism in the *Ras-Related Protein Rab-24* (*RAB24*) gene, a member of the RAS oncogene family, which encodes a protein necessary for autophagosome trafficking, was found responsible for juvenile onset ataxia in some breeds (Agler et al., 2014). A missense mutation in the *autophagy related 4D cysteine peptidase* (*ATG4D*) gene was linked to vacuolar storage deficiency and neurodegeneration in Lagotto Romagnolo dogs (Kyöstilä et al., 2015). A study investigating juvenile onset neuroaxonal dystrophy in Spanish Water Dogs identified a non-synonymous mutation in the *tectonin beta-propeller repeat containing 2* (*TECPR2*) gene, which had been linked to autophagosome formation (Hahn et al., 2015). A very similar type of neuroaxonal dystrophy exists in humans; hence, this finding could have actually suggested a possible genetic background to look for in affected people.

DM is another example of a naturally occurring neurodegenerative disease in dogs and shows a high degree of similarity to human ALS. Both DM and ALS have been linked to mutations in the ROS neutralizing *SOD1* gene, suggesting a shared genetic and metabolic background. Importantly, the possible contribution of autophagy to motor neuron loss was reported to be controversial both in DM (Ogawa et al., 2015) and in ALS (Chen et al., 2012). Autophagy also has a similarly controversial role in muscular atrophy in humans and dogs (Sandri, 2010; Pagano et al., 2015). Altogether, these findings indicate many homologies between dogs and humans regarding the regulation of autophagy in aging and disease.

### Deregulation of Nutrient Sensing

**Figure 6:**
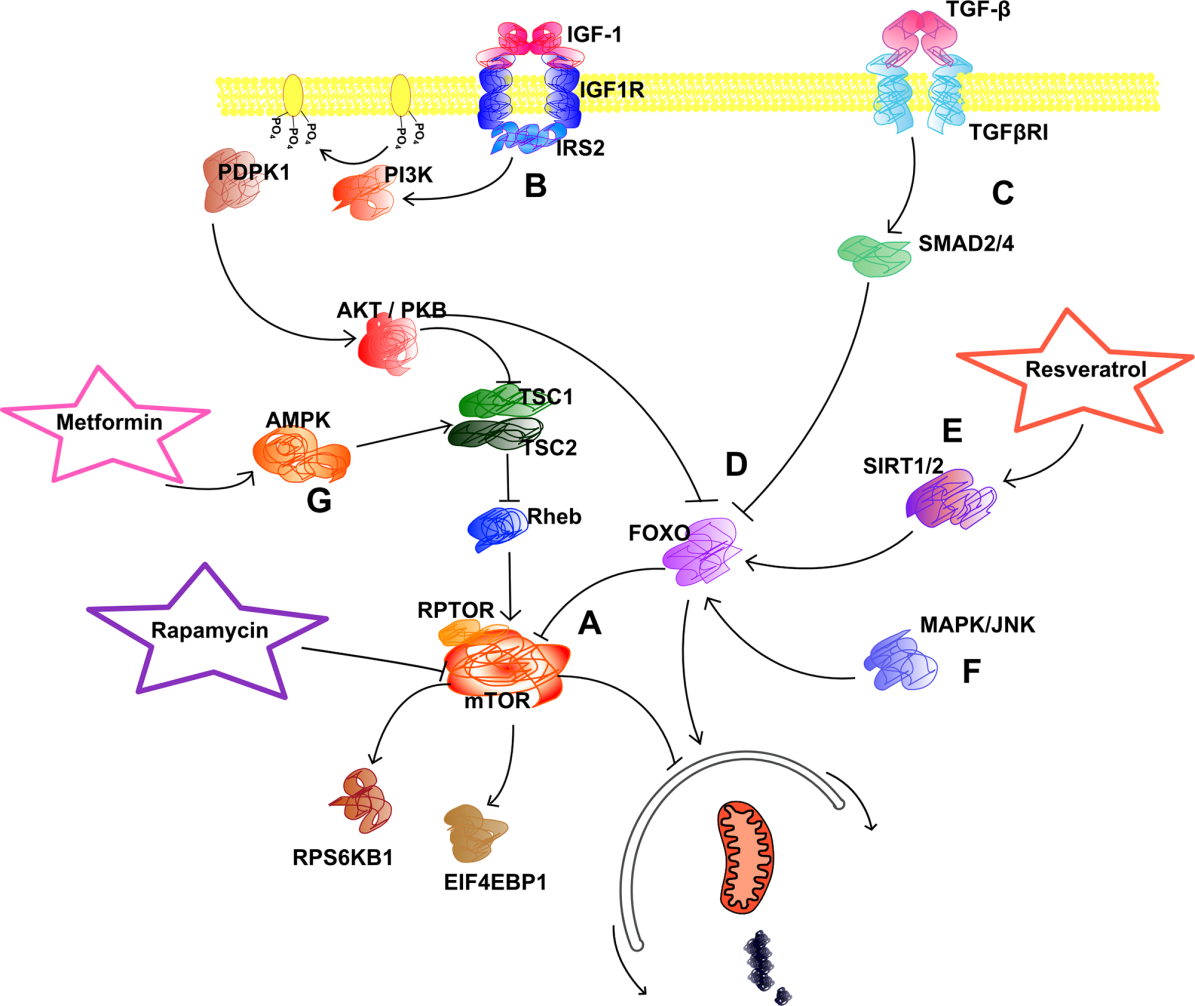
Signaling pathways. This figure illustrates some of the many signaling pathways that have been connected to aging. Activating interactions are shown with arrows, while inhibiting interactions are represented by bar headed lines. **(A)** Almost all of the age-related signaling pathways converge on the metabolic signal integrator mTORC1 complex, which includes the mTOR kinase together with RPTOR and other proteins. mTORC1 integrates stimuli to fine-tune metabolic processes, protein synthesis, cell growth, and autophagy. Downstream targets of mTORC1 include ribosomal proteins and translation initiation factors, like RPS6KB1 and EIF4EBP1, as well as ULK1, which is an activator of autophagy. As its name indicates, mTOR is the main target of rapamycin, which inhibits its function. **(B)** The IGF1 signaling is considered to be the main modulator that links autophagy to aging. Upregulation of this pathway leads to repression of autophagy and activation of protein synthesis by mTOR. This pathway includes many proteins, most of which have kinase activity. The PI3K enzymes transmit the signal from the IGF1 receptor by phosphorylating phosphatidylinositol molecules in the membrane, which then activate PDPK1. From here, the signal is forwarded to AKT (also known as PKB) by phosphorylation. AKT then inhibits the function of the TSC1 and TSC2 proteins, and consequently releases RHEB from inhibition. RHEB directly binds and activates the mTORC1 complex. **(C)** Another signaling pathway, which acts parallel to IGF1, is the TGF-β signalization. It is implicated in cellular growth control and also in tumorigenesis and inhibits autophagy. SMAD proteins transduce the TGF-β signal to downstream targets. An important target of SMAD2/4 is the FOXO gene family. **(D)** FOXO transcription factors have an evolutionary conserved function in aging regulation and integrate several pathways to upregulate autophagy and inhibit mTORC1. **(E)** Sirtuins (SIRT1/2) act contrary to the TGF-β pathway as they upregulate FOXO and thus autophagy. Resveratrol and caloric restriction exert their anti-aging effect through the activation of sirtuins. **(F)** The MAPK proteins were also shown to play a role in aging by regulating FOXO. They serve as important early responsive elements of different cellular stimuli and also play a role in apoptotic cell death induction in the case of UV-light damage. **(G)** AMPK integrates metabolite sensing information and acts contrary to the IGF1 pathway: activation of AMPK leads to down-regulation of mTOR and activation of autophagy. AMPK is the main target of metformin. mTOR, mechanistic target of rapamycin; RPTOR, regulatory associated protein of MTOR; RPS6KB1, ribosomal protein S6 kinase, 70kD, polypeptide 1; EIF4EBP1, eukaryotic translation initiation factor 4E binding protein 1; ULK1, unc-51 like autophagy activating kinase 1; IGF1, insulin-like growth factor 1; PIK3, phosphatidylinositol-4,5-bisphosphate 3-kinase; PDPK1, 3-phosphoinositide dependent protein kinase 1; AKT, AKT serine/threonine kinase 1; TSC1, tuberous sclerosis 1; TSC2, tuberous sclerosis 2; RHEB, Ras homolog, mTORC1 binding protein b; complex 1; TGF-β, transforming growth factor β; SMAD, MAD, mothers against decapentaplegic; FOXO, forkhead box O; SIRT, sirtuin; MAPK, mitogen-activated protein kinase; AMPK, adenosine monophosphate kinase.

Cellular metabolism, protein synthesis, and autophagy are strictly regulated by various signaling pathways (**Figure 6**) (Martindale and Holbrook, 2002; He and Klionsky, 2009). Most of these have evolved to synchronize cell growth and metabolism with nutrient availability; hence, they are often referred to as nutrient sensing pathways. Many of them converge on the target of rapamycin (TOR) kinase (**Figure 6A**), a main factor in determining rates of protein turnover and metabolism (Wullschleger et al., 2006). In mammals, the TOR kinase may function in different complexes, named mTORC1 or mTORC2, depending on its protein partners. The mTORC1 complex, which includes the regulatory associated protein of MTOR (RPTOR) protein, corresponds to the invertebrate TOR complex in its regulatory interactions, while mTORC2 controls other intracellular processes. Knock-down of TOR expression by RNA interference was shown to increase lifespan of *C. elegans* by threefold (Vellai et al., 2003). Later, similar effects of inhibiting TOR or its homologs were reported in *S. cerevisiae* (Kaeberlein et al., 2005), *D. melanogaster* (Kapahi et al., 2004), and laboratory mice (Wu et al., 2013), emphasizing its conserved role in the aging process (Kapahi et al., 2010).

Importantly, the function of mTOR can be efficiently inhibited by rapamycin, which is an already approved immunosuppressant in human medicine, and therefore has been proposed as a promising anti-aging compound to be used in humans. However, it was reported to cause severe side effects in medical dosages (Hartford and Ratain, 2007). Therefore, optimal dosages, which do not cause undesirable syndromes, yet still exert longevity promoting effects should be carefully determined in preclinical studies. Actually, pharmaceutical studies have already been initiated to investigate the effects of rapamycin on the lifespan of dogs (Kaeberlein et al., 2016; Urfer et al., 2017).

One of the main signaling pathways that regulate TOR activity is the insulin and IGF1 signaling (IIS) pathway (**Figure 6B**). It was first linked to aging when strains of *C. elegans* with doubled lifespan revealed a mutation in *daf-2*, the worm homologue of the *IGF1 receptor* (*IGF1R*) gene (Kenyon et al., 1993). Later, a threefold elongation in the non-replicative lifespan of *S. cerevisiae* was also linked to two genes that functioned in the glucose sensing pathway (Thevelein and de Winde, 1999). In flies and mice with hypomorphic alleles of *IGF1R* homologs, a significant increase in lifespan was observed together with characteristic pleiotropic effects (Tatar et al., 2001; Holzenberger et al., 2003). However, the longevity effect was less pronounced in mice, pointing at the possibility that the relative contribution of IIS to aging regulation may differ in various taxa.

Importantly, comparisons between centenarian and younger human cohorts also showed associations between expected lifespan and serum IGF1 levels or genetic polymorphisms in related genes (Barbieri et al., 2003; Van Heemst et al., 2005). Furthermore, functional variants in *IGF1R* were shown to be enriched in centenarians (Suh et al., 2008). Polymorphisms in other genes of the IIS pathway were also linked to longevity in GWAS, although not without contradictions (Soerensen et al., 2012a). In fact, some studies reported a decrease in GH and IGF1 plasma levels during normal aging (Breese et al., 1991; Sonntag et al., 1997). In this regard, it was hypothesized that the reduction in IGF1 levels may actually serve as a first line compensatory mechanism when age-related damage starts to accumulate in cells (Schumacher et al., 2008; López-Otín et al., 2013). Although low basic IIS signaling may delay aging, the overcompensation resulting from continuously accumulating damage in aged individuals may lead to insufficiencies in IGF1 signaling, and this can cause further decline. This effect may be particularly relevant in the case of neural aging, because the brain has special metabolic properties and a high need for optimal glucose levels. In support of this hypothesis, some age-related neurodegenerative states were linked to low IGF1 levels (Sonntag et al., 2000; Sonntag et al., 2005; Moloney et al., 2010). In addition, lower plasma IGF1 levels were shown to impair vascular maintenance in the brain (Sonntag et al., 1997). In this regard, dogs, which are more similar to humans in brain physiology and function than rodents, also seem promising to further unfold the relationship between IGF1 signaling and healthy aging.

Importantly, functional mutations in the *IGF1* and *IGF1R* genes have already been linked to body size variability in dogs (Sutter et al., 2007; Hoopes et al., 2012; Rimbault et al., 2013), similarly to humans and laboratory animals (Liu et al., 1993; Ishida et al., 1998; Perry and Dominy, 2009). The notion that small dog breeds usually live longer than large breeds (Galis et al., 2007; Greer et al., 2007; Kraus et al., 2013) also hints at the potential role of the IIS pathway in canine lifespan determination. In fact, the genomic region harboring the *IGF1* locus was linked to size and lifespan across different breeds (Jones et al., 2008) and serum IGF1 levels were shown to correlate with age and obesity (Greer et al., 2011) in individual dogs.

Some other pathways linked to body size in dogs (Rimbault et al., 2013; Schoenebeck and Ostrander, 2014) are also known to regulate TOR and autophagy. The *SMAD family member 2* (*SMAD2*) gene, which functions in the transforming growth factor beta (TGF-β) pathway (**Figure 6C**) (Vellai, 2009), was associated with body size (Rimbault et al., 2013) and was previously found to be in linkage with mortality of dog breeds (Jones et al., 2008). The *growth hormone receptor* (*GHR*) and *growth* hormone (*GH*) genes, which also modulate dogs’ body size, were shown to affect longevity in humans (Soerensen et al., 2012a; van der Spoel et al., 2016), and in mice (Bartke et al., 2001; Flurkey et al., 2001; Kinney et al., 2001; Amador-Noguez et al., 2004).

Both IIS and TGF-β signaling have several targets beyond TOR, and many of them were implicated in aging. For example, the forkhead box O (FOXO) transcription factors are targeted by both IGF1 and TGF-β signaling (**Figure 6D**) and were shown to have an important role in tumor suppression (Greer and Brunet, 2005) and age-related diseases (Hesp et al., 2015). The worm homologue of the mammalian *FOXO* genes, *daf-16*, was one of the first genes linked to extreme longevity in *C. elegans*, as it was found necessary for the longevity effect observed in *daf-2* deficient worms (Kenyon et al., 1993; Larsen et al., 1995; Lin et al., 1997; Ruvkun et al., 1997). Out of the four mammalian orthologues, *FOXO3a* has been associated with aging in human cohort studies (Willcox et al., 2008; Anselmi et al., 2009; Soerensen et al., 2010). *FOXO1a* SNPs were also reported to affect longevity, however, in a gender-specific manner (Li et al., 2009). In addition, FOXO3 was shown to regulate autophagy in skeletal muscle and plays a role in muscular atrophy (Mammucari et al., 2007). Despite the emphasized role of *FOXOs* in disease and aging, the canine homologues have not yet been studied in detail.

Sirtuins (**Figure 6E**) and the 5’ AMP-activated protein kinase (AMPK) also play an important role in nutrient sensing. Sirtuins function as nicotinamide adenine dinucleotide (NAD) dependent protein deacetylases, sensing the levels of NAD in cells. Decreased NAD levels were shown to reduce their activity, and thus NAD replacement therapies have been suggested as possible anti-aging interventions (Chini et al., 2017). AMPK detects the levels of adenosine monophosphate (AMP) in cells, and it is able to counteract IGF1 signaling by inhibiting TOR (Inoki et al., 2003; Gwinn et al., 2008) and activating FOXO3a (Sanchez et al., 2012). It was also suggested to play a major neuroprotective role by activating the UNC-51 Like 1 (ULK1) kinase (Kim et al., 2011), a key autophagy inducing protein (Wong et al., 2013).

Both sirtuins and AMPK are targets of several proposed anti-aging drugs. For example, the anti-diabetic drugs, metformin and acarbose, were both shown to exert an anti-aging effect in model animals (Anisimov et al., 2011; Cabreiro et al., 2013; Martin-Montalvo et al., 2013; Harrison et al., 2014) and to modulate various aging-related intracellular processes, including the activation of AMPK (Cho et al., 2015; Lu et al., 2015; Chan et al., 2016). Importantly, metformin was actually reported to decrease mortality of diabetic people in comparison to patients treated with other drugs in a retrospective large-scale study (Bannister et al., 2014). Consequently, metformin has been suggested as a promising candidate for anti-aging interventions (Barzilai et al., 2016). It may also be easily applied in family dogs to test its longevity enhancing potential, because it was already shown to have relatively mild side effects in dogs (Heller, 2007).

However, it is important to note that all of these drugs, including rapamycin, may exert pleiotropic effects in organisms through various cellular signaling and regulatory mechanisms. For example, metformin was implicated to modify the composition of the gut microbiome in diabetic patients (Forslund et al., 2015), which in turn can indirectly affect aging and neural function (see below in the section *Microbiome*). Such pleiotropic effects should be thoroughly considered in humans and family dogs, as both are exposed to variable environmental stimuli, have diverse genetic background, and may use other medications, which can alter the mechanisms of actions of anti-aging compounds through complex interactions.

A fairly recently emerged possible regulator of aging that interacts with IGF1 signaling and FOXO activity is the klotho hormone, which was first identified in mice as a longevity factor (Kuro-o et al., 1997; Kurosu et al., 2005). The *klotho* (*KL*) gene represents an example of longevity genes that are missing from invertebrate models, but show functional polymorphisms associated with human longevity (Arking et al., 2002).

### Mitochondrial Dysfunction

**Figure 7:**
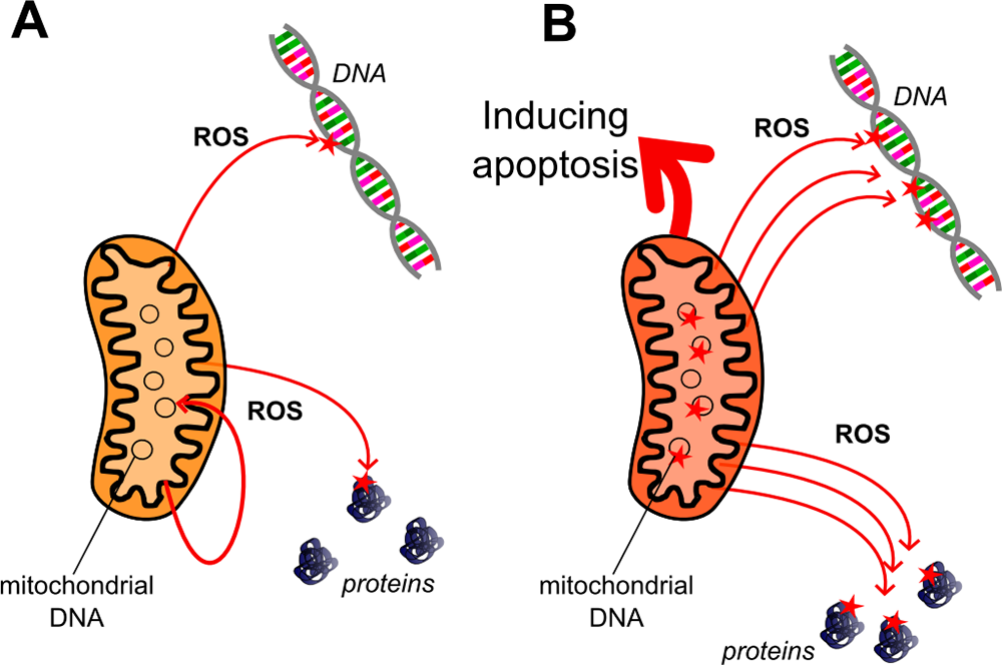
Mitochondria and oxidative stress. **(A)** Mitochondria represent the main source for reactive oxygen species (ROS) within eukaryotic cells as the oxidative respiration processes take place in the inner membrane of mitochondria, utilizing a special electron transport chain. Increased respiration rate due to metabolic changes and reduced antioxidant accessibility may also increase generation of ROS, which can damage the mitochondrial genome as well (indicated by thick arrow). **(B)** Accumulation of mutations in the mitochondrial genome may lead to malfunction in the electron transport chain in aberrant mitochondria that consequently produce an elevated rate of ROS. Removal of aberrant mitochondria is a key process for maintaining oxidative balance in cells.

Nutrient sensing pathways converge on the regulation of mitochondrial activity, as these organelles are the main sources of energy (in the form of adenosine triphosphate, ATP) in eukaryotic cells under normal circumstances, when enough oxygen is present. The availability of nutrients determines the rate of mitochondrial respiration, which, however, generates not only ATP but also chemical by-products, including ROS (**Figure 7A**). The oxidative burden created by mitochondria may be especially high in neurons, which solely depend on aerobic mitochondrial respiration as energy source. In accordance with this, associations between chronological age and a higher ROS production rate of mitochondria in brain cells were demonstrated in rodents (Navarro and Boveris, 2004; Petrosillo et al., 2008), humans (Mecocci et al., 1993), and also in dogs (Head et al., 2009). Oxidative DNA lesions could also occur in the mitochondrial genome and consequently modify gene expression and optimal function of mitochondria, possibly leading to a positive feedback mechanism in the generation of extensive levels of ROS. In fact, age-related changes in mitochondrial gene expression profiles were reported in mice (Manczak et al., 2005) and the accumulation of mitochondrial DNA mutations, mainly deletions, in certain brain regions was linked to impaired mitochondrial respiration in humans (Corral-Debrinski et al., 1992; Kraytsberg et al., 2006). Importantly, mitochondrial dysfunction is considered to be a main driver of the pathophysiology of neurodegenerative diseases, including Alzheimer’s disease, Parkinson’s disease, and ALS (Lin and Beal, 2006). For example, gain-of-function mutations in the *Leucine-rich repeat kinase 2* (*LRRK2*) gene causing autosomal dominant form of PD (Di Fonzo et al., 2005) were shown to result in hampered mitochondrial function (Mortiboys et al., 2010). Although the generation of malfunctioning mitochondria may be counterbalanced by elevated levels of mitophagy, a form of macroautophagy that is able to degrade mitochondria, it is not yet clear in the literature how mitophagic activity is generally changed in affected cells of PD patients (Chu, 2019).

The role of mitochondrial dysfunction and increased oxidative burden in neural aging was also investigated in dogs. In general, dog brains were shown to accumulate oxidative damage with age (for more details, see the section *Genomic Instability*). In a study published by Head et al., 2009, mitochondrial ROS production and complex I driven respiration rate showed significant alterations between old and young laboratory Beagle dogs from the same colony, with aged dogs having higher ROS production and lower respiration rate. The same study reported that a diet enriched with antioxidants and mitochondrial cofactors improved mitochondrial respiration rate and reduced ROS production in aged dogs, and also had a positive effect on their cognitive performance. In another study, a ketosis-inducing diet was shown to modify mitochondrial function and, to some extent, reduce amyloid-β deposition in dog brains (Studzinski et al., 2008). Interestingly, Christie et al. reported in 2009 that short-term supplementation with lipoic acid (LA), which is both an important mitochondrial cofactor and a powerful antioxidant, did not improve cognitive function of aged Beagle dogs, contrary to their previous findings where LA had been used together with other antioxidants (Cotman et al., 2002; Milgram et al., 2002a; Milgram et al., 2002b). Furthermore, another mitochondrial cofactor, acetyl-L-carnitine (ALCAR), when supplemented in itself, was shown to decrease cognitive performance (Christie et al., 2009). In a paper, Snigdha et al., 2016 who also failed to replicate findings about the beneficial effects of antioxidant enriched diet on the cognition of aged Beagle dogs, suggested that these controversies could have resulted from differences in the baseline nutrition of dogs.

Nevertheless, such findings could also result from a more complicated interaction between ROS and aging, as it was suggested by several authors, who re-evaluated the classical theories about the connection between mitochondria, ROS, and general longevity, based on the increasing body of experimental evidences (Hekimi et al., 2011; López-Otín et al., 2013). Accordingly, the ambiguous results listed above could result from altered mitochondrial homeostasis (mithormesis) in cells and not solely from impaired mitochondrial respiration and ROS overproduction. The concept of mithormesis suggests that mild mitochondrial stressors may actually benefit cellular health and longevity (López-Otín et al., 2013). Such minor stress, which, for example, can be induced by pharmaceutical agents, can boost mitochondrial turnover and activate defensive mechanisms (Haigis and Yankner, 2010). As it was shown that malfunctioning mitochondria can also directly affect aging by other mechanisms than increased oxidative stress (Trifunovic et al., 2004; Vermulst et al., 2008; Edgar et al., 2009; Hiona et al., 2010), for example by inducing apoptosis (Kroemer et al., 2007) (**Figure 7B**), elevated mitochondrial turnover can actually protect cells from these deleterious effects. Since both resveratrol and metformin have been hypothesized to be mild mitochondrial toxins, they may also exert their anti-aging effect at least partly by inducing mithormesis (López-Otín et al., 2013).

Altogether, mitochondria-targeting interventions may require mindful considerations, especially in populations with high genetic variability. Genetic variants in mitochondrial genomes are known to cause disorders in humans (Koopman et al., 2012), and they may also substantially alter the capability of cells to cope with mitochondrial poisons (Finsterer and Frank, 2016). This means that interactions between mitochondrial genotypes and specific chemical compounds should also be considered in anti-aging intervention studies. In this regard, dogs can again become ideal models with good translatability. Several mitochondrial diseases are known in dogs, which have human homologs, such as the sensory ataxic neuropathy found in Golden Retriever dogs (Baranowska et al., 2009) or the familial dilated cardiomyopathy in Doberman Pinschers (Meurs et al., 2012). As several promising anti-aging drugs are likely to be tested in dogs in preclinical studies, looking into their effects on mitochondrial function and testing their possible interactions with mitochondrial genotypes can be highly relevant for humans.

### Cellular Senescence

All the mechanisms discussed so far act congruently to modulate cellular metabolism, growth, proliferation, and, eventually, senescence, which is characterized by a permanent cell cycle arrest and stereotyped phenotypic changes (Campisi and d’Adda di Fagagna, 2007; Collado et al., 2007). Specific mechanisms serve as effectors of senescence in response to unrepaired DNA damage, mitochondrial malfunction, and other forms of excessive stress. Most famously, telomere attrition represents a somewhat genetically programmed route to cellular senescence as it was implied from experiments with human fibroblasts (Harley et al., 1990; Allsopp et al., 1992). However, the fact that cultured murine cells also reached senescence despite their telomerase positivity suggested that other mechanisms, like increased oxidative damage in cultured conditions, should have contributed to their limited proliferative potential (Sherr and DePinho, 2000).

Among other examples, the activation of the p16^INK4a^/Rb and the p14^ARF^/p53 signaling pathways is able to induce cellular senescence in response to time-dependent changes, including the accumulation of DNA damage (d’Adda di Fagagna, 2008). The expression of the *INK4/ARF* locus, which encodes the p16^INK4^, p15^INK4^, and p14^ARF^ proteins in humans, was reported to correlate with chronological age in various tissues of rodents and humans (Krishnamurthy et al., 2004; Krishnamurthy et al., 2006; Ressler et al., 2006; Liu et al., 2009b), nominating it as an ideal biomarker of aging. Importantly, the *INK4/ARF* locus can directly affect healthspan and longevity, as it was shown experimentally in mice (Matheu et al., 2009). Furthermore, simultaneous overexpression of *INK4/ARF* and *p53* caused extended lifespan in mice, accompanied by cancer resistance and reduced neural decline (Matheu et al., 2007; Carrasco-Garcia et al., 2015). The human *INK4/ARF* locus was also found to be the most strongly associated locus with several age-related pathologies in a meta-analysis of GWAS (Jeck et al., 2012), and it was associated with longevity in a smaller cohort study (Emanuele et al., 2010). Interestingly, the elevated expression and activity of p53 in itself did not increase longevity in experimental studies (García-Cao et al., 2002; Mendrysa et al., 2006). Actually, certain hyperactive variants of p53 were shown to reduce lifespan in mice, although with simultaneously prompting increased cancer resistance (Tyner et al., 2002; Dumble et al., 2007).

In this regard, it is important to note that the p14/p53 pathway, together with p16/pRb, are fundamental tumor suppressor mechanisms; therefore, they unquestionably contribute to healthy aging by forcing potentially malignant cells into a senescent state or into programmed cell death (Hickman et al., 2002).

This anti-tumor effect of induced cellular senescence can explain the contradictory findings regarding the role of p53 and other tumor suppressor mechanisms in aging (Rodier and Campisi, 2011), as a possible trade-off exists between longevity and cancer occurrence (Matheu et al., 2008).

So far, no studies have investigated the canine homologs of the *INK4/ARF* locus and *p53* in relation to aging. However, and not surprisingly, their role and regulation in tumorigenesis showed high similarities between dogs and humans (Rowell et al., 2011; Lutful Kabir et al., 2013), suggesting that their roles in aging, especially in healthy aging, are also conserved in dogs.

#### Accumulation of Senescent Cells in Tissues

The time-dependent upregulation of senescence inducing mechanisms means that the ratio of aged cells may gradually increase in the tissues of older individuals. Indeed, a marked elevation of senescent cell numbers was reported in old mice (Wang et al., 2009), although not in all tissues. Importantly, this accumulation process can result from both the increased generation of senescent cells and a decreased activity of macrophages that are able to eliminate aged or apoptotic cells from tissues. As the activity of the innate immune system was shown to decrease with age (Plowden et al., 2004; Mahbub et al., 2011), it is likely that reduced phagocytic capacity also contributes to organismal aging through the disrupted elimination of senescent cells (López-Otín et al., 2013). Furthermore, senescent cells were shown to produce inflammatory signals and create a special inflammatory microenvironment around themselves (Kuilman et al., 2010; Rodier and Campisi, 2011), which may directly contribute to tissue aging by creating a positive feedback loop and inducing cellular senescence in neighboring cells (Nelson et al., 2012). This so-called “bystander” effect was actually shown to modulate the number of senescent cells *in vivo* in mice (da Silva et al., 2019).

Little is known about the accumulation of senescent cells in canine tissues, although this phenomenon is also likely to show fundamental similarities with other mammalian species. As there is a growing interest toward pharmacological approaches to deplete senescent cells in tissues by specific apoptosis inducing agents (senolytic drugs) (Kirkland et al., 2017), dogs may eventually be involved in testing these types of anti-aging interventions.

### Stem Cell Exhaustion

Tissue renewal depends on the abundance and replicative capacity of tissue-specific stem cells, which can replace cells lost by terminal senescence or apoptosis. Thus, the age-related increase in cellular senescence may also result in elevated stem cell activation and differentiation, eventually causing the depletion of stem cell pools. In fact, early exhaustion of stem cells in certain tissues was shown to accelerate aging in flies and mice (Cheng et al., 2000; Kippin et al., 2005; Rera et al., 2011). Furthermore, it was shown that increased basic fibroblast growth factor (FGF2) signaling in muscle tissues of aged mice accelerated depletion of stem cells by forcing them to leave quiescent state (Chakkalakal et al., 2012).

Importantly, senescence may also directly affect stem cells, depriving them from the ability to replicate and differentiate even if they are still present in tissues. For example, hematopoietic stem cells (HSCs) were reported to have reduced replicative capacity in both aged mice and humans, mainly because of accumulating DNA damage (de Haan and Lazare, 2018). This reduction can explain the old age anemia of elderly people (Patel, 2008). Importantly, similar forms of age-associated changes in blood parameters, including anemia, were reported in dogs (Strasser et al., 1993; Radakovich et al., 2017).

Stem cell quiescence and activation is regulated by many of the already discussed aging pathways, including p53 and IIS (Liu et al., 2009a; Xian et al., 2012). Thus, pharmacological interventions that act on these could also affect stem cell dynamics. In this regard, the inhibition of mTOR was shown to have beneficial effects on aging by promoting cellular rejuvenation (Castilho et al., 2009; Chen et al., 2009; Yilmaz et al., 2012). Furthermore, pharmacological inhibitors of the Cell Division Cycle 42 (CDC42) protein, which is an inducer of HSC senescence, were shown to promote rejuvenation of HSC pools in mice (Florian et al., 2012).

Besides pharmacological interventions, stem cell therapy has also been suggested as a possible anti-aging intervention, with highlighted promises to treat certain forms of neurodegeneration (Lindvall et al., 2004; Trounson and DeWitt, 2016). In this regard, stem cell therapy trials conducted on dogs affected with CCD or other forms of neurodegeneration could represent a crucial step before progressing to human trials. In the case of the Golden Retriever model for Duchenne muscular dystrophy, successful stem cell-based interventions had actually preceded human clinical trials (Pelatti et al., 2016). Other instances of dog stem cell therapy trials were discussed by Hoffman and Dow (2016).

### Altered Intercellular Communication

In the course of evolution, several mechanisms have evolved to establish efficient communication between cells in multicellular organisms. Intercellular communication types include paracrine, endocrine, and neurocrine signaling, and all of these can be involved in the aging process. Especially hormones and other endocrine signal transducers can have a main role in systemic aging regulation. Actually, GH and the insulin/IGF1 signaling pathway belong to these main systemic regulators, most of which are supervised by the hypothalamic–pituitary–adrenal (HPA) and –thyroid axes. Endocrine signaling also involves hormones synthetized by the digestive system and reproductive glands. Furthermore, small molecules produced by gut bacteria can also have systemic effects on the host organism (Donia and Fischbach, 2015).

#### Neuroendocrine Signaling

The CNS mainly functions as conductor, coordinating various processes of the organism according to intrinsic and extrinsic stimuli. Signals provided by the CNS—together with the digestive system—can affect every part of the body. In this regard, “neural aging” has recently gained more focus as a central mechanism, which could impact the systemic aging of the whole organism (Weir and Mair, 2016). In support of this theory, both neuronal and intestinal genetic manipulations, which reduced mitochondrial electron transport chain function, were shown to extend lifespan in *C. elegans*, while similar manipulations in other tissues had no longevity effect (Durieux et al., 2011).

Importantly, several signaling pathways have been hypothesized to play fundamental roles in both neural senescence and systemic aging. For example, IGF1, together with the brain-derived neurotrophic factor (BDNF) and serotonin, were shown to affect brain aging and modulate metabolic changes linked to caloric restriction across the body (Mattson et al., 2004). The hypothalamus also has major implications in aging. For example, reproductive aging was shown to be controlled by the gonadotropin releasing hormone (GnRH), which is produced by special cells in the hypothalamus (Yin and Gore, 2006). Age-related reduction in GnRH levels, in response to activation of inflammatory pathways, was suggested to aggravate frailty and neurodegeneration in the elderly (Zhang et al., 2013). Altogether, age-related changes in the hypothalamus and, consequently, in HPA regulation seem to play a central role in the systemic regulation of aging (Deuschle et al., 1997; Kim and Choe, 2019). The activity of the hypothalamus and the HPA axis was reported to show similar general attributes in dogs as in humans and age-related changes in the HPA axis were already assessed in dogs (Reul et al., 1991; Rothuizen et al., 1991). However, further studies will be needed to investigate the function of GnRH and other hormones in canine aging.

#### Parabiosis Experiments and Systemic Factors of Aging

Most molecular effectors of systemic aging are excreted into the blood, by which they can reach every part of the body. This mediatory function of the blood was proven by parabiosis experiments in rodents, when the artificial connection of the circulatory systems of old and young animals resulted in beneficial effects on the cognitive performance of aged individuals (Katsimpardi et al., 2014; Villeda et al., 2014). Several of the possible effector molecules behind this phenomenon have been revealed since (Loffredo et al., 2013; Demontis et al., 2014; Elabd et al., 2014). Interestingly, some of the systemic factors present in human umbilical cord plasma were shown to beneficially influence brain aging when applied experimentally in mice (Castellano et al., 2017), indicating conserved functions for these molecules. It is important to note, however, that other blood-borne factors were shown to actually promote aging. For example, the β2 microglobulin was reported to negatively affect cognitive performance and regenerative potentials in aged mice (Smith et al., 2015). Although parabiosis is not really applicable in humans and in family dogs, the identified systemic factors seem promising as effectors or targets for anti-aging interventions in both species and may be introduced to preclinical studies conducted on dogs.

#### Extracellular Vesicles

In addition to hormones and metabolites, extracellular vesicles released by cells into the blood, called exosomes and ectosomes, have emerged as important transducers of various cellular signals (Meldolesi, 2018) and their content, including miRNAs, may provide diagnostic and prognostic measures for many diseases, including AD (Cheng et al., 2014; Cheng et al., 2015; Thind and Wilson, 2016; Van Giau and An, 2016). Consequently, exosomes may also modulate aging and neurodegeneration (Cheng et al., 2015). In support of this, it was recently demonstrated by Zhang et al. (2017) that the stem cells of the hypothalamus could affect the speed of aging by exosomal miRNAs secreted into the cerebrospinal fluid in mice.

Exosome research in dogs have been limited until recently. However, blood miRNA levels—which were hypothesized to be mainly found in exosomes—were reported to correlate with disease phenotypes in canine Duchenne muscular dystrophy (Mizuno et al., 2011). Similarly, miRNA content in circulating exosomes was shown to correlate with progression of secondary heart failure in cases of myxomatous mitral valve disease in dogs (Yang et al., 2017). Direct links were suggested between alterations in urinary exosome formation, miRNA content, and occurrence of kidney disease in dogs by Ichii et al., 2017. Furthermore, a recent study reported exosome derived miRNAs as biomarkers for canine mammary tumors (Fish et al., 2018). Altogether, investigations about the connections between exosome content and aging or age-related pathologies in dogs may lead to the identification of diagnostic markers with potential translational prospects into human studies.

#### Immunaging and Inflamm-Aging

Together with the CNS, the immune system has a main systemic regulatory function in the organism. Most immune cells synthesize various signaling molecules that act either in a paracrine or endocrine manner and can also provide defense against various pathogens. Macrophages, which are part of the innate immune system, can wander throughout the body and have important roles in tissue homeostasis by cleaning cellular debris and pathogens.

The human immune system is known to experience a general age-related decline in its function and in the abundance of some cell types, although the exact details of reported changes may vary between studies (Pawelec, 2018). In general, reduced numbers of naïve CD8+ T cells and moderately elevated numbers of memory T cells were found to be linked to aging. Importantly, bone marrow derived macrophages were also shown to lose phagocytic capacity with aging (Kim et al., 2017; Li et al., 2017), which can contribute to the accumulation of senescent cells in tissues.

In addition, it has long been hypothesized that systemic age-related changes in certain immune components linked to inflammation will lead to a so-called inflamm-aging phenomenon. Importantly, the exact interactions between immunosenescence and inflamm-aging have not yet been clarified (Fulop et al., 2018); hence, further studies using systems biology approaches may shed light on the detailed mechanisms that underlie them (Ostan et al., 2017).

Both immunosenescence and inflamm-aging were proposed as contributors to aging and age-related pathologies in dogs (Day, 2010). Large-scale hematologic and serum phenotyping studies done in various breeds (Faldyna et al., 2001; Lawrence et al., 2013; Chang et al., 2016) showed that several of the assessed blood parameters correlated with chronological age. Regarding the immune system, T and B lymphocytes were mainly affected in most cases; however, the directions of these changes were contradictory (Faldyna et al., 2001; Massimino et al., 2003; HogenEsch et al., 2004; Reis et al., 2005; Greeley et al., 2006). However, a recent study reported that changes in naïve and memory T cell numbers in old dogs were similar to those previously described in most human studies (Withers et al., 2018). Importantly, it was shown that lifelong calorie restriction positively affected lymphocyte numbers in aged dogs (Greeley et al., 2006). Taken together, the dog may become one of the most applicable model animals to study immunosenescence and inflamm-aging, and to test interventions that could attenuate the deterioration of the immune system.

#### Microbiome

Recent findings suggest that both systemic metabolism and immune function can be modulated by bacteria inhabiting the gut, termed gut microbiome (Tremaroli and Bäckhed, 2012). The microbiome can interact with the host organism through various chemical signals, and some of these may directly affect the function of distant organs, like the brain (Sharon et al., 2016). Therefore, the microbiome may fundamentally affect health and disease, and possibly aging (Zapata and Quagliarello, 2015). This was supported by findings that reported consistent changes in the composition of the microbiome in elderly people and centenarians (Biagi et al., 2010; Biagi et al., 2017). Although these correlations do not necessarily indicate causative links (Saraswati and Sitaraman, 2015), the theory of microbial modulation of aging has been gaining more and more scientific interest. Importantly, experimental evidences from rodents have already shown that the microbiome can affect the progression of neurodegeneration (Sampson et al., 2016). Furthermore, probiotics and prebiotics, which can beneficially alter the composition of the microbiome, were reported to positively influence the aging of the gut and systemic inflammation in people (Patel et al., 2014; Vaiserman et al., 2017).

Currently, not much is known about age-related changes in the canine gut microbiome; however, there is growing resear ch interest in this field. Future findings may have direct implications to humans as well, because the composition of the canine microbiome was shown to be more similar to humans than that of mice and pigs (Coelho et al., 2018) and actual correlations between the microbiomes of dogs and people living in the same household were also reported (Misic et al., 2015). Because dogs age faster than humans, they can be ideal models to test the potential aging effects of prebiotics and probiotics in longitudinal follow-up studies. Importantly, some probiotics used in humans were already suggested to promote health in dogs (Grześkowiak et al., 2015), and this can facilitate their adaptation for systemic anti-aging intervention trials.

It is important to note that other microbial niches on the human body may also affect aging and disease, as it was implicated when oral microbiota and inflammation were linked to the progression of Alzheimer’s disease (Pritchard et al., 2017). As periodontitis is also a serious health issue in aged dogs (Albuquerque et al., 2012), this link between oral microbiome and neurodegeneration undoubtedly requires further focus in canine aging research and veterinary medicine and may also benefit humans by translational studies (An et al., 2018).

## Conclusions and Perspectives

**Figure 8:**
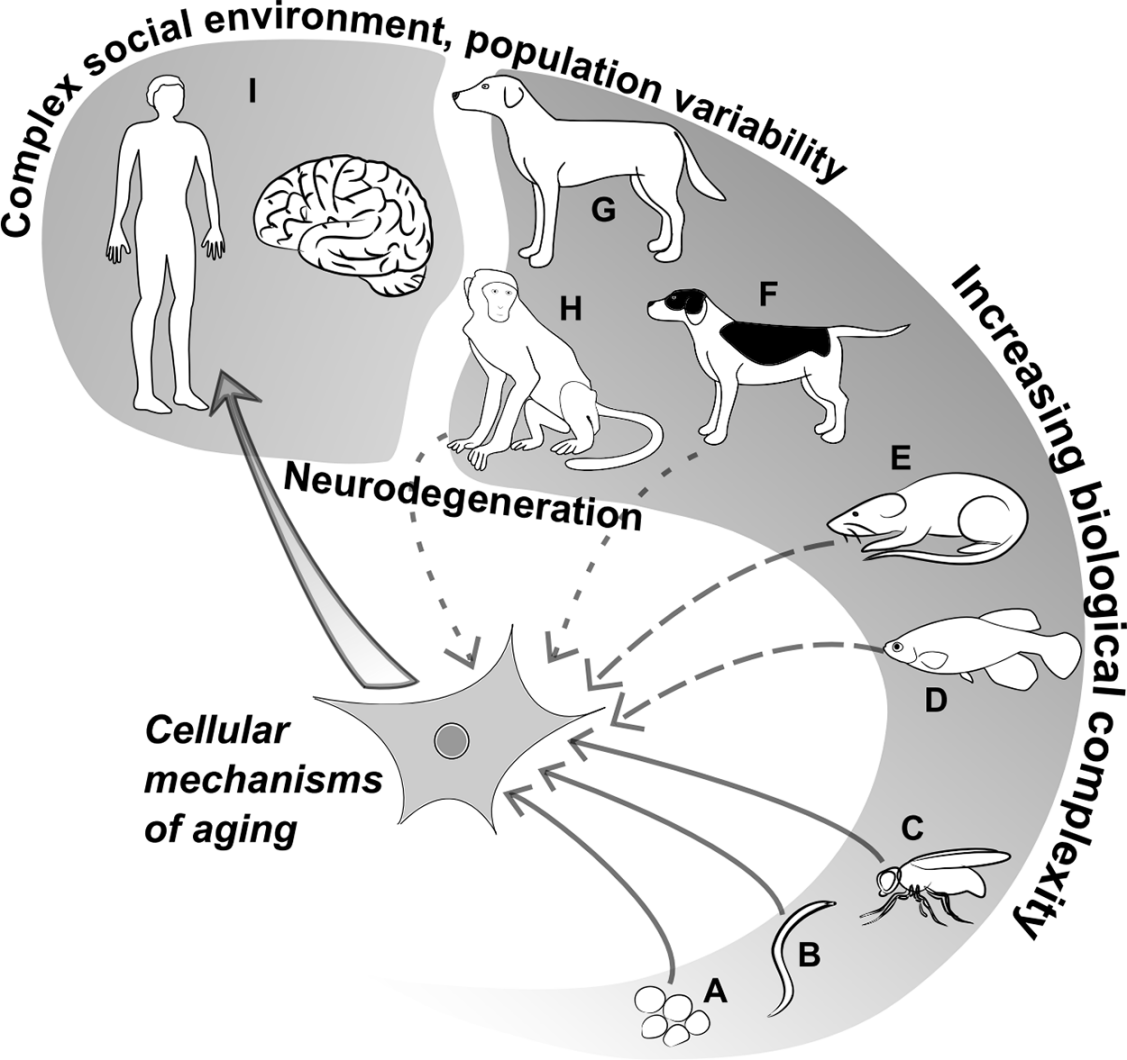
Model organisms of aging. The figure illustrates common aging model organisms, including small animal models and large aninmal models used to study various aspetcs of aging. **(A**–**C)** Yeast (*Saccharomyces cerevisiae*) and the invertebrates *Caenorhabditis elegans* and *Drosophila melanogaster* are ideal to experimentally study the basic, conserved mechanisms of cellular – and organismal – aging. On the other hand, they show less biological complexity than vertebrates in many aspects, and they do not naturally develop neurodegeneration. **(D**–**E)** Vertebrate small animal models, like the turqoise killifish (*Nothobranchius furzeri*) and rodents (*Mus musculus* and *Rattus norvegicus*) are ideal to study the biological mechanisms that may be absent in invertebrates, and they can still be rather easily used in experimental studies, including genetic manipulations. However, they typically do not develop age-related neurodegeneration, and may lack many aspects of the complex social and environmental influencers of human aging. **(F**–**G)** Dogs show similarities to humans in their physiology and they tend to naturally develop age-related cognitive decline. Laboratory dogs **(F)** are traditional large animal models in pharmacology reserach. However, the same way as other laboratory models, they do not represent the natural genetic and environmental variability typical for human populations. Family dogs, **(G)** on the other hand, live in the same environment as humans do, and show a special population genetic stratification, with the presence of genetically isolated, diverse populations (breeds). **(H)** Primates are the closest related to humans, thus they may seem to be the most appropriate animals to study human aging. However, primates are not suited for large-scale sudies for many reasons, including ethical and financial ones. Although they tend to develop human-like age-related neurodegeneration, they still lack the genetic and environmental complexity (both in the laboratory and in their natural habitats), which may influence human aging phenotypes in human populations. **(I)** Human aging shows many unique attributes, including a high prevalence of neurodegeneration. Age-related neurodegeneration is hard to study in most animals, and translational experiments have had many limitations so far. Brain aging may be fundamentally affected by non-genetic factors, including diet, exercise and social environment, which seem challenging to be modelled under laboratory conditions to reflect the natural circumstances of human populations.

Considering the remarkably complex nature of biological processes that underlie aging, it is not surprising that finding a biological model for aging that would unify all relevant aspects is challenging. Family dogs have been proposed as ideal models to complement findings from other model organisms (**Figure 8**); however, the still limited knowledge about the exact genetic and regulatory mechanisms that underlie their aging may restrict their applicability in translational studies. Although searching for links between genetic variants and aging phenotypes would be more challenging than in the case of other phenotypic parameters, which are easier to measure, such approaches seem indispensable to gain insight into the main genetic mechanisms that modulate aging variability in dogs. Actually, there have been some efforts along these lines (Jones et al., 2008); however, interbreed comparisons have a limited potential to reveal the exact variants responsible for longevity differences between individuals. Future studies should aim at intrabreed approaches, for which both genetic and aging related data should be available from the same animals. Furthermore, gene expression mapping could be a novel approach to pinpoint at pathways that show changes between young and old dogs or between dogs with short and long lifespan. Although it could be challenging to obtain good quality tissues from a large number of family dogs with known lifespan and other parameters, biobanks created following human examples may help to overcome this limitation in the long term.

As recent findings have increased the palette of possible anti-aging interventions, making almost all of the nine hallmarks of aging (López-Otín et al., 2013) targetable by drugs, a growing interest for preclinical testing of these compounds is expected. Consequently, the dog may gain more and more attention as a preclinical model species. Because family dogs are exposed to almost the same background effects, which can modify the outcome of interventions, as are people, they might even become inevitable to provide a suitable model to assess the effects of anti-aging interventions on natural populations.

It is important to emphasize that characterizing the aging process of dogs and establishing effective interventions within the species may benefit humans not only by clarifying scientific questions but also by making it possible to increase the healthy lifespan of companion and service animals. Owning a guide dog or service dog can lead to great improvements in the quality of life of disabled people. Also, service dogs may facilitate human–human interactions and contribute to the socio-emotional well-being of their owners. Caron-Lormier et al. (2016) reported that most guide dogs were retired due to age-related diseases or simply old age, after an average of 8.5 years of service. Increasing the lifespan and healthspan of working dogs could be emotionally beneficial for their owners, and also could be financially advantageous for societies, as the training of these animals is time consuming and expensive. Furthermore, providing average family dogs an elongated healthspan may also benefit their owners. Several studies have reported a positive correlation between dog walking, physical activity, and health variables in owners, although results were often controversial, suggesting the need for further research on this topic (Brown and Rhodes, 2006; Lentino et al., 2012; Christian et al., 2018). In some cases, improvements were most pronounced in older cohorts (Thorpe et al., 2006; Toohey et al., 2013; Garcia et al., 2015; Curl et al., 2016). Experiences from animal-assisted therapy also suggested that animal–human interactions may help the elderly to experience a successful aging course (Baun and Johnson, 2010). Therefore, providing a long and healthy life for companion animals may benefit the health and welfare of their owners as well.

Taken together, strong scientific evidence suggests that utilizing dogs as models of human aging and anti-aging interventions may hold prospects unattainable by other model organisms, if the complex interactions between genetics and environmental factors are taken into consideration. Thus, canine studies on aging may bring forward results that can eventually benefit the elderly as well as their pets.

## Author Contributions

SS collected literature and wrote manuscript parts regarding genetic pathways in aging and in dogs. EK collected literature and wrote manuscript parts regarding dogs as models in general, environmental, and cognitive factors. Both authors worked on reviewing the final text. EK provided funding.

## Funding

This project has received funding from the European Research Council (ERC) under the European Unions Horizon 2020 research and innovation program (Grant Agreement No. 680040), the János Bolyai Research Scholarship of the Hungarian Academy of Sciences, the Bolyai+ ÚNKP-18-4 New National Excellence Program of the Ministry of Human Capacities, and the Hungarian Brain Research Program 2017-1.2.1-NKP-2017-00002.

## Conflict of Interest

The authors declare that the research was conducted in the absence of any commercial or financial relationships that could be construed as a potential conflict of interest.
