# Supplementary material for: Genetic Pathways of Aging and Their Relevance in the Dog as a Natural Model of Human Aging
Source: Front Genet. 2019 Oct 18;10:948. doi: 10.3389/fgene.2019.00948 (PMC6813227; doi:10.3389/fgene.2019.00948)
Supplement: Supplementary file 1 [file DataSheet_1.pdf]

Supplementary material

Sándor, S., & Kubinyi, E. (2019). Genetic pathways of aging and their relevance in the dog as a natural model of human aging. Frontiers in Genetics, 10, 948. doi: 10.3389/FGENE.2019.00948

https://www.frontiersin.org/articles/10.3389/fgene.2019.00948/abstract

Beyond genetics

Complexity behind aging

Together with the complex network of genetic components discussed in the main text, environmental factors, including diet, exercise, stress, pollution and socio-economic environment also make a fundamental impact on human aging. Actually, the heritability of individual differences in aging phenotypes and average lifespan did not exceed 35% in many studies (Crimmins and Finch, 2012; Finch and Tanzi, 1997). (However, the heritability of extreme longevity was found to be higher – see e.g. Perls et al. (1998) and Sebastiani and Perls (2012)). Revealing the key environmental elements that modify expected lifespan and untangling their complex interactions with the genetic background should be an important step to develop effective interventions. In this regard, experimental models may have severe limitations, because many of the factors that affect natural human populations are challenging to be mimicked under laboratory conditions, or are simply not relevant in model animals because of their limited social capacities. In this aspect the dog has unmatched potentials, because its social and physiological needs closely resemble those of humans. There are empirical and also scientific indications

that training and socialization level can affect the well-being and health status of companion dogs (Arhant et al., 2010; Blackwell et al., 2008; Hiby et al., 2004).

Exercise

In modern societies both humans and their pets are affected by a so called “westernized” lifestyle, which is characterized by dramatically reduced physical activity, an increase in psychological stress and high calorie intake. These factors are considered responsible for an elevated rate of obesity and increased incidence of cardiovascular disease and cancer, which are known to share molecular mechanisms with aging. Therefore, it seems a valid assumption that such lifestyle changes would decrease average lifespan, However, it is likely that the concurrent developments in medical care systems may have actually confounded these effects in humans - and companion animals (Bonnett and Egenvall, 2010). Yet, an increasing body of evidence suggests that health parameters can be severely hindered by the lack of exercise. Physical exercise was shown to reduce anxiety and depression, improve cognitive function and decrease the occurrence and severity of certain diseases and neurodegeneration, both in humans and mice (Churchill et al., 2002; Eriksson and Gard, 2011; Hillman et al., 2008; Kronenberg et al., 2006; Larson et al., 2006; Salmon, 2001). It was also shown to slow down the progression of Alzheimer’s disease or other forms of cognitive decline and muscular atrophy (Adlard et al., 2005; Ahlskog et al., 2011; Heyn et al., 2004) (Cartee et al., 2016).

The negative behavioral and health consequences of reduced exercise in dogs are likely to be similar, although not many studies have investigated this question so far (Kobelt et al., 2007). In the future, systematic exercise interventions, together with diet changes and behavioral enrichment may yield positive results in dogs as well.

Diet

The composition of one's diet has long been considered a key element in health and disease. In modern human societies obesity is one of the main health concerns (Williams et al., 2015) which – in addition to low levels of daily exercise - is mainly caused by consuming high-fat, high-calorie diet, often called "westernized" diet (Cordain et al., 2005). Obesity is also prevalent in pet animals (German, 2006). Physiological changes in obese dogs show similar patterns as in humans, proposing the species as a large animal model of obesity (Kleinert et al., 2018; Osto and Lutz, 2015; Stachowiak et al., 2016). Importantly, obesity was reported to shorten healthspan and lifespan in laboratory dogs and in pet dogs (German, 2006; Salt et al., 2018). Also, laboratory dogs kept on a restricted diet lived longer and healthier than paired animals kept on a normal diet (Larson et al., 2003). These findings were in concordance with the vast evidence that supported the longevity effects of restricted diet regimens in several other species. The most comprehensively investigated dietary intervention, called caloric restriction (CR), consists of a low calorie diet, which still contains the optimal amount of essential biomolecules, minerals and vitamins. It was first shown to extend lifespan of rats and mice (McCay et al., 1935; Weindruch and Walford, 1982) and later the same beneficial effects were reported from other examples, including yeast (Lin et al., 2000) worms (Klass, 1977) and even non-human primates (Colman et al., 2009, 2014), suggesting an evolutionary conserved physiological basis for this effect. Importantly, CR was also reported to be effective even when implemented in old animals (Cao et al., 2001). Although it is still a fundamental question, whether the increase in healthspan and lifespan would reach the same extent in humans, case reports have already confirmed a potential longevity effect for various forms of diet restrictions in isolated human populations (Willcox et al., 2006). Furthermore, voluntary participants who followed a strict diet for years were reported to have lower activity of the insulin signaling pathway and their gene expression profiles changed towards a pattern typical for younger people (Mercken et al., 2013). Another study reported improved muscular mitochondrial function in response to CR in young adult humans (Civitarese et al., 2007). Importantly, both CR and intermittent fasting were shown to be an effective

intervention to delay or even reverse cognitive decline and slow down the progression of Alzheimer's disease (Luchsinger et al., 2002; Patel et al., 2005; Witte et al., 2009). All these effect can result from the concordant modulation of several age-related pathways by CR. IGF1 signaling (Kari et al., 1999), the activation of sirtuins (Cohen et al., 2004) and the epigenetic inhibition of somatic transposon activity (De Cecco et al., 2013) were all shown to be affected by nutritional status. On the other hand, the universality of CR as a powerful tool to increase life- and healthspan has been questioned by several studies that showed no effect or even negative effects of restricted food intake in rodents, depending on their genetic background (Barrows and Roeder, 1965; Fernandes et al., 1976; Forster et al., 2003). A more comprehensive study further supported these findings by screening heterogeneous populations of mice for the effects of caloric restriction (Liao et al., 2010). Later, Schleit et al. (2013) pinpointed various possible molecular pathways that may be at the bottom of such differences. Nevertheless, it is important to note, that the type of dietary restriction may vary among studies, and thus not all conditions of restricted diet may correspond with the exact definition of caloric restriction. Yet, these findings clearly demonstrated the need for more research on the applicability of CR in highly variable natural populations. Studies on human populations, however, are not easy to be conducted, especially when the effects of lifelong CR are to be evaluated. In this regard, the dog can again become a favorable model system, as a species with high genetic variability, coupled with lifestyle and diet variation between individuals. Longitudinal studies on laboratory Beagle and Labrador retriever dogs have already shown promising results regarding the applicability of dietary interventions (Kealy et al., 2002; Larson et al., 2003; Wang et al., 2007), yet it is important to note, that physiological responses to caloric restriction may vary among breeds, similarly as it was reported in mice. For this reason, further studies should be designed to investigate the effects of CR on family dogs, which represent a much wider range of breeds.

References

Adlard, P. A., Perreau, V. M., Pop, V., and Cotman, C. W. (2005). Voluntary Exercise Decreases Amyloid Load in a Transgenic Model of Alzheimer's Disease. *J. Neurosci.* 25. Available at: http://www.jneurosci.org/content/25/17/4217.short [Accessed April 21, 2017].

Ahlskog, J. E., Geda, Y. E., Graff-Radford, N. R., and Petersen, R. C. (2011). Physical Exercise as a Preventive or Disease-Modifying Treatment of Dementia and Brain Aging. *Mayo Clin. Proc.* 86, 876–884. doi:10.4065/mcp.2011.0252.

Arhant, C., Bubna-Littitz, H., Bartels, A., Futschik, A., and Troxler, J. (2010). Behaviour of smaller and larger dogs: Effects of training methods, inconsistency of owner behaviour and level of engagement in activities with the dog. *Appl. Anim. Behav. Sci.* 123, 131–142. doi:10.1016/j.applanim.2010.01.003.

Barrows, C. H., and Roeder, L. M. (1965). *The effect of reduced dietary intake on enzymatic activities and life span of rats.* Gerontological Society by C.C. Thomas Available at: https://www.cabdirect.org/cabdirect/abstract/19651403826 [Accessed May 19, 2018].

Blackwell, E. J., Twells, C., Seawright, A., and Casey, R. A. (2008). The relationship between training methods and the occurrence of behavior problems, as reported by owners, in a population of domestic dogs. *J. Vet. Behav. Clin. Appl. Res.* 3, 207–217. doi:10.1016/j.jveb.2007.10.008.

Bonnett, B. N., and Egenvall, A. (2010). Age Patterns of Disease and Death in Insured Swedish Dogs , Cats and Horses. *J. Comp. Pathol.* 142, S33–S38. doi:10.1016/j.jcpa.2009.10.008.

Cao, S. X., Dhahbi, J. M., Mote, P. L., and Spindler, S. R. (2001). Genomic profiling of short- and long-term caloric restriction effects in the liver of aging mice. *Proc. Natl. Acad. Sci. U. S. A.* 98, 10630–5. doi:10.1073/pnas.191313598.

Cartee, G. D., Hepple, R. T., Bamman, M. M., and Zierath, J. R. (2016). Exercise Promotes Healthy

Aging of Skeletal Muscle. *Cell Metab.* 23, 1034–1047. doi:10.1016/J.CMET.2016.05.007.

Churchill, J. D., Galvez, R., Colcombe, S., Swain, R. A., Kramer, A. F., and Greenough, W. T. (2002). Exercise, experience and the aging brain. *Neurobiol. Aging* 23, 941–955. doi:10.1016/S0197-4580(02)00028-3.

Civitarese, A. E., Carling, S., Heilbronn, L. K., Hulver, M. H., Ukropcova, B., Deutsch, W. A., et al. (2007). Calorie Restriction Increases Muscle Mitochondrial Biogenesis in Healthy Humans. *PLoS Med.* 4, e76. doi:10.1371/journal.pmed.0040076.

Cohen, H. Y., Miller, C., Bitterman, K. J., Wall, N. R., Hekking, B., Kessler, B., et al. (2004). Calorie Restriction Promotes Mammalian Cell Survival by Inducing the SIRT1 Deacetylase. *Science (80-.).* 305. Available at: http://science.sciencemag.org/content/305/5682/390 [Accessed June 23, 2017].

Colman, R. J., Anderson, R. M., Johnson, S. C., Kastman, E. K., Kosmatka, K. J., Beasley, T. M., et al. (2009). Caloric Restriction Delays Disease Onset and Mortality in Rhesus Monkeys. *Science (80-.).* 325. Available at: http://science.sciencemag.org/content/325/5937/201 [Accessed April 19, 2017].

Colman, R. J., Beasley, T. M., Kemnitz, J. W., Johnson, S. C., Weindruch, R., and Anderson, R. M. (2014). Caloric restriction reduces age-related and all-cause mortality in rhesus monkeys. *Nat. Commun.* 5, 3557. doi:10.1038/ncomms4557.

Cordain, L., Eaton, S. B., Sebastian, A., Mann, N., Lindeberg, S., Watkins, B. A., et al. (2005). Origins and evolution of the Western diet: health implications for the 21st century. *Am. J. Clin. Nutr.* 81, 341–354. doi:10.1093/ajcn.81.2.341.

Crimmins, E. M., and Finch, C. E. (2012). The Genetics of Age-Related Health Outcomes. *Journals Gerontol. Ser. A Biol. Sci. Med. Sci.* 67A, 467–469. doi:10.1093/gerona/gls101.

De Cecco, M., Criscione, S. W., Peterson, A. L., Neretti, N., Sedivy, J. M., and Kreiling, J. A. (2013). Transposable elements become active and mobile in the genomes of aging mammalian somatic tissues. *Aging (Albany. NY).* 5, 867–83. doi:10.18632/aging.100621.

Eriksson, S., and Gard, G. (2011). Physical exercise and depression. *Phys. Ther. Rev.* 16, 261–268. doi:10.1179/1743288X11Y.0000000026.

Fernandes, G., Yunis, E. J., and Good, R. A. (1976). Influence of diet on survival of mice. *Proc. Natl. Acad. Sci. U. S. A.* 73, 1279–83. doi:10.1073/PNAS.73.4.1279.

Finch, C. E., and Tanzi, R. E. (1997). Genetics of Aging. *Science (80-.).* 278. Available at: http://science.sciencemag.org/content/278/5337/407/tab-pdf [Accessed April 21, 2017].

Forster, M. J., Morris, P., and Sohal, R. S. (2003). Genotype and age influence the effect of caloric intake on mortality in mice. *FASEB J.* 17, 690–692. doi:10.1096/fj.02-0533fje.

German, A. J. (2006). The Growing Problem of Obesity in Dogs and Cats. *J. Nutr.* 136, 1940S-1946S. doi:10.1093/jn/136.7.1940S.

Heyn, P., Abreu, B. C., and Ottenbacher, K. J. (2004). The effects of exercise training on elderly persons with cognitive impairment and dementia: A meta-analysis. *Arch. Phys. Med. Rehabil.* 85, 1694–1704. doi:10.1016/j.apmr.2004.03.019.

Hiby, E., Rooney, N., and Bradshaw, J. (2004). Dog training methods : their use , effectiveness and interaction with behaviour and welfare. *Anim. Welf.* 13, 63–69. Available at: https://www.researchgate.net/profile/Nicola_Rooney/publication/261106650_Dog_training_methods_Their_use_effectiveness_and_interaction_with_behaviour_and_welfare/links/542e8ada0cf27e39fa962554/Dog-training-methods-Their-use-effectiveness-and-interaction-with-behaviour-and-welfare.pdf [Accessed April 21, 2017].

Hillman, C. H., Erickson, K. I., and Kramer, A. F. (2008). Be smart, exercise your heart: exercise effects on brain and cognition. *Nat. Rev. Neurosci.* 9, 58–65. doi:10.1038/nrn2298.

Kari, F. W., Dunn, S. E., French, J. E., and Barrett, J. C. (1999). Roles for insulin-like growth factor-1 in mediating the anti-carcinogenic effects of caloric restriction. *J. Nutr. Health Aging* 3, 92–101. Available at: http://www.ncbi.nlm.nih.gov/pubmed/10885804 [Accessed June 23, 2017].

Kealy, R. D., Lawler, D. F., Ballam, J. M., Mantz, S. L., Biery, D. N., Greeley, E. H., et al. (2002). Effects of diet restriction on life span and age-related changes in dogs. *J. Am. Vet. Med. Assoc.* 220, 1315–1320. doi:10.2460/javma.2002.220.1315.

Klass, M. R. (1977). Aging in the nematode Caenorhabditis elegans: Major biological and environmental

factors influencing life span. *Mech. Ageing Dev.* 6, 413–429. doi:10.1016/0047-6374(77)90043-4.

Kleinert, M., Clemmensen, C., Hofmann, S. M., Moore, M. C., Renner, S., Woods, S. C., et al. (2018). Animal models of obesity and diabetes mellitus. *Nat. Rev. Endocrinol.* 14, 140–162. doi:10.1038/nrendo.2017.161.

Kobelt, A. J., Hemsworth, P. H., Barnett, J. L., Coleman, G. J., and Butler, K. L. (2007). The behaviour of Labrador retrievers in suburban backyards: The relationships between the backyard environment and dog behaviour. *Appl. Anim. Behav. Sci.* 106, 70–84. doi:10.1016/j.applanim.2006.07.006.

Kronenberg, G., Bick-Sander, A., Bunk, E., Wolf, C., Ehninger, D., and Kempermann, G. (2006). Physical exercise prevents age-related decline in precursor cell activity in the mouse dentate gyrus. *Neurobiol. Aging* 27, 1505–1513. doi:10.1016/j.neurobiolaging.2005.09.016.

Larson, B. T., Lawler, D. F., Spitznagel, E. L., and Kealy, R. D. (2003). Improved Glucose Tolerance with Lifetime Diet Restriction Favorably Affects Disease and Survival in Dogs. *J. Nutr.* 133, 2887–2892. doi:10.1093/jn/133.9.2887.

Larson, E. B., Wang, L., Bowen, J. D., McCormick, W. C., Teri, L., Crane, P., et al. (2006). Exercise Is Associated with Reduced Risk for Incident Dementia among Persons 65 Years of Age and Older. *Ann. Intern. Med.* 144, 73. doi:10.7326/0003-4819-144-2-200601170-00004.

Liao, C.-Y., Rikke, B. A., Johnson, T. E., Diaz, V., and Nelson, J. F. (2010). Genetic variation in the murine lifespan response to dietary restriction: from life extension to life shortening. *Aging Cell* 9, 92–95. doi:10.1111/j.1474-9726.2009.00533.x.

Lin, S.-J., Defossez, P.-A., and Guarente, L. (2000). Requirement of NAD and SIR2 for Life-Span Extension by Calorie Restriction in Saccharomyces cerevisiae. *Science (80-.).* 289. Available at: http://science.sciencemag.org/content/289/5487/2126 [Accessed April 20, 2017].

Luchsinger, J. A., Tang, M.-X., Shea, S., Mayeux, R., A, H., and M, B. (2002). Caloric Intake and the Risk of Alzheimer Disease. *Arch. Neurol.* 59, 1258. doi:10.1001/archneur.59.8.1258.

McCay, C. M., Crowell, M. P., and Maynard, L. A. (1935). The effect of retarded growth upon the length of life span and upon the ultimate body size. *J. Nutr.* 5, 155–171. Available at:

http://www.wealthandhealth.ltd.uk/over100/C. M. McCAY 1935.pdf [Accessed April 30, 2018].

Mercken, E. M., Crosby, S. D., Lamming, D. W., JeBailey, L., Krzysik-Walker, S., Villareal, D. T., et al. (2013). Calorie restriction in humans inhibits the PI3K/AKT pathway and induces a younger transcription profile. *Aging Cell* 12, 645–651. doi:10.1111/acel.12088.

Osto, M., and Lutz, T. A. (2015). Translational value of animal models of obesity—Focus on dogs and cats. *Eur. J. Pharmacol.* 759, 240–252. doi:10.1016/J.EJPHAR.2015.03.036.

Patel, N. V., Gordon, M. N., Connor, K. E., Good, R. A., Engelman, R. W., Mason, J., et al. (2005). Caloric restriction attenuates Aβ-deposition in Alzheimer transgenic models. *Neurobiol. Aging* 26, 995–1000. doi:10.1016/j.neurobiolaging.2004.09.014.

Perls, T. T., Bubrick, E., Wager, C. G., Vijg, J., and Kruglyak, L. (1998). Siblings of centenarians live longer. *Lancet (London, England)* 351, 1560. doi:10.1016/S0140-6736(05)61126-9.

Salmon, P. (2001). Effects of physical exercise on anxiety, depression, and sensitivity to stress: A unifying theory. *Clin. Psychol. Rev.* 21, 33–61. doi:10.1016/S0272-7358(99)00032-X.

Salt, C., Morris, P. J., Wilson, D., Lund, E. M., and German, A. J. (2018). Association between life span and body condition in neutered client-owned dogs. *J. Vet. Intern. Med.* 33, jvim.15367. doi:10.1111/jvim.15367.

Schleit, J., Johnson, S. C., Bennett, C. F., Simko, M., Trongtham, N., Castanza, A., et al. (2013). Molecular mechanisms underlying genotype-dependent responses to dietary restriction. *Aging Cell* 12, 1050–1061. doi:10.1111/acel.12130.

Sebastiani, P., and Perls, T. T. (2012). The genetics of extreme longevity : lessons from the New England Centenarian study. *Front. Genet.* 3, 1–7. doi:10.3389/fgene.2012.00277.

Stachowiak, M., Szczerbal, I., and Switonski, M. (2016). Genetics of Adiposity in Large Animal Models for Human Obesity—Studies on Pigs and Dogs. *Prog. Mol. Biol. Transl. Sci.* 140, 233–270. doi:10.1016/BS.PMBTS.2016.01.001.

Wang, Y., Lawler, D., Larson, B., Ramadan, Z., Kochhar, S., Holmes, E., et al. (2007). Metabonomic Investigations of Aging and Caloric Restriction in a Life-Long Dog Study. *J. Proteome Res.* 6,

1846–1854. doi:10.1021/PR060685N.

Weindruch, R., and Walford, R. (1982). Dietary restriction in mice beginning at 1 year of age: effect on life-span and spontaneous cancer incidence. *Science (80-.).* 215. Available at: http://science.sciencemag.org/content/215/4538/1415/tab-pdf [Accessed April 19, 2017].

Willcox, D. C., Willcox, B. J., Todoriki, H., Curb, J. D., and Suzuki, M. (2006). Caloric restriction and human longevity: what can we learn from the Okinawans? *Biogerontology* 7, 173–177. doi:10.1007/s10522-006-9008-z.

Williams, E. P., Mesidor, M., Winters, K., Dubbert, P. M., and Wyatt, S. B. (2015). Overweight and Obesity: Prevalence, Consequences, and Causes of a Growing Public Health Problem. *Curr. Obes. Rep.* 4, 363–370. doi:10.1007/s13679-015-0169-4.

Witte, A. V, Fobker, M., Gellner, R., Knecht, S., and Flöel, A. (2009). Caloric restriction improves memory in elderly humans. *Proc. Natl. Acad. Sci. U. S. A.* 106, 1255–60. doi:10.1073/pnas.0808587106.
